# Experimental and Mesoscale Numerical Investigation of Fracture Behavior in MICP-Cemented Sand Under Different Loading Conditions

**DOI:** 10.3390/ma19204413

**Published:** 2026-10-20

**Authors:** Yue Dai, Mayao Cheng, Xinhua Yang, Peifeng Huang, Qin Qian

**Affiliations:** 1 School of Civil Engineering and Transportation, Foshan University, Foshan 528225, China; daiyue9611@sina.com (Y.D.); 2112211022@stu.fosu.edu.cn (P.H.); 2 School of Aerospace Engineering, Huazhong University of Science and Technology, Wuhan 430074, China; qqian@hust.edu.cn

**Keywords:** MICP-cemented sand, multiphase particle placement, cohesive zone model (CZM), numerical-experimental comparison, crack propagation paths

## Abstract

**Highlights:**

**What are the main findings?**

- A multiphase algorithm places sand and CaCO_3_ polymorphs explicitly.
- A cohesive zone model (CZM)-based mesoscale FE model captures matrix cracking and interfacial debonding.
- Tension, shear, and compression show distinct macroscopic failure modes.

**What are the implications of the main findings?**

- The framework links particle-scale packing to interface-dominated fracture in MICP sand.
- Weak interfaces and heterogeneous precipitates control crack paths and macroscopic failure modes.
- The 2D model applies mainly to low-confinement, monotonic loading conditions.

**Abstract:**

Microbially induced calcium carbonate precipitation (MICP) can transform a loose sand mass into a cemented porous medium. To elucidate its deformation and failure behavior, we developed a two-dimensional mesoscale finite element model. The model integrates a novel multiphase random particle placement algorithm with a cohesive zone model (CZM) to explicitly represent the heterogeneous microstructure of sand grains and CaCO_3_. The model was used to simulate crack evolution and mechanical response under uniaxial tension, compression, and direct shear, with the results validated against experimental data. Under uniaxial compression, the simulated peak strength was approximately 2.65 MPa, compared with an experimental average of 2.53 MPa. The proposed model accurately represents the multiphase microstructure and captures the main mechanical response characteristics. The results show that the sand-grain arrangement has a limited influence on peak strength but significantly affects post-peak behavior by controlling crack propagation. The simulations reproduced distinct failure modes: brittle tensile rupture, quasi-brittle shear failure, and mixed quasi-brittle-frictional compressive failure with inclined shear bands. Across all loading conditions, a common mesoscale failure mechanism was identified: microcracks initiate in weak or stress-concentrated regions, propagate along CaCO_3_-sand interfaces, and coalesce into through-going fractures. This study enhances the understanding of mechanics of MICP-cemented sand and provides a mechanism-oriented mesoscale framework for future analyses.

## 1. Introduction

Microbially induced calcium carbonate precipitation (MICP) is an environmentally friendly, in situ ground improvement technique with tunable-structure modification capabilities, showing considerable potential in sand stabilization, fracture sealing and subsurface remediation [1]. MICP is a biomineralization process driven by ureolytic bacteria and other microorganisms, in which bacterial metabolism promotes CaCO_3_ precipitation at particle contacts and along pore walls, forming bridging structures [2]. This process restructures the internal load-transfer network of the soil, thereby modifying porosity and permeability and enhancing macroscopic mechanical properties [3]. As CaCO_3_ precipitates accumulate, they fill the pore space and form cementing bridges, causing the sand skeleton to gradually evolve from a frictional granular assembly to a cemented porous medium [4]. Grain contacts change from predominantly geometric, frictional contacts to CaCO_3_-bonded cohesive contacts, and the mechanical response shifts from that of an unstructured sand to a rock-like porous skeleton [5,6].

Extensive physical experiments on MICP have been conducted in recent years. However, due to the complexity of the biomineralization process and the multitude of influencing factors, experimental results often exhibit significant scatter and occasional contradictions, making it difficult to derive unified guidelines for engineering practice [7]. The biochemical reactions of MICP [2,8] are governed by coupled microbial and chemical conditions, including bacterial strain and metabolic pathway [9,10], cell concentration and activity [11,12], pH and buffer system [13], temperature [14], calcium source type [15], Ca^2+^ and urea concentrations and their ratio [12,16], regulating ions [17], nutrient composition [18], as well as soil pore size [19] and particle size distribution [20]. These parameters collectively control the spatial distribution, polymorph, volume fraction and interface properties of the MICP precipitates, which in turn strongly influence the macroscopic mechanical behavior of the cemented material [8,21]. Existing studies have mainly focused on tailoring process parameters to improve the strength and stiffness of MICP-treated soils [8]. Since the microstructure and composition of the precipitates can be manipulated via process control, clarifying the intrinsic links between microstructure and mechanical performance is of great importance for advancing MICP toward robust engineering applications.

Although physical testing most accurately reflects real boundary and loading conditions, it suffers from long testing periods, high cost, limited repeatability, and difficulties in strictly isolating single variables, which poses challenges for revealing the underlying micromechanical mechanisms [22]. Numerical modeling, as an important complement to laboratory testing, has attracted increasing attention due to its efficiency, controllability, and rich data output. However, it remains in an early stage for MICP systems [23]. Existing numerical approaches for predicting the mechanical behavior of MICP-cemented materials can be broadly grouped into three categories: (1) Machine-learning-based models [24,25], which use large experimental datasets to train predictive models for strength and other properties. These models exhibit good fitting and generalization capability, but are essentially data-driven “black boxes” that provide limited insight into the causal relationships between microstructure and macroscopic behavior. (2) Discrete element method (DEM) models [26,27], which are well suited to capturing particle contacts and rearrangements at the grain scale. However, in many studies, CaCO_3_ precipitation is simplified as an equivalent bond or as modified contact parameters, making it difficult to realistically represent the morphology, spatial heterogeneity, and multiphase nature of the precipitates, thereby limiting a deeper analysis of fracture paths and energy dissipation mechanisms [28]. (3) Continuum-scale coupled models [29,30,31], which adopt a bio-chemical-hydraulic-mechanical (BCHM) framework to describe the injection, transport, and reaction of biogrouts in saturated, deformable porous media. While these models are powerful for simulating reaction-transport processes, the mechanical response of the cemented skeleton is often treated in an equivalent manner, with insufficient attention paid to strength evolution and fracture mechanisms governed by the detailed microstructure of the precipitates [29]. In summary, current modeling approaches tend to emphasize either data-driven prediction, grain-scale contact mechanics, or coupled reaction-transport processes, and to varying degrees neglect the key microstructural features of MICP products, namely their multiphase, heterogeneous, and morphologically variable nature [32]. There is thus a clear need for models that can explicitly represent the microstructure of the precipitates and faithfully simulate their fracture process to more reliably investigate the mechanical performance of MICP-cemented materials.

Experimental evidence shows that MICP precipitates constitute a multiphase composite composed of crystalline phases such as calcite, aragonite, and vaterite [33,34,35], together with amorphous calcium carbonate, bacterial bodies, and other amorphous components [36,37]. A key scientific challenge is therefore how to accurately characterize this multiphase, strongly heterogeneous structure at the mesoscale and reliably predict the macroscopic mechanical response under different stress conditions, including tension, compression, and shear [38]. To address this issue, the present study develops a mesoscale finite element modeling approach that combines multiphase random particle placement with a cohesive zone model (CZM). In this framework, sand grains and multiple CaCO_3_ polymorphs (calcite, aragonite, and vaterite) are explicitly placed as discrete particles, while CZM elements are introduced to represent damage and fracture along interfaces within the amorphous matrix and between the matrix and the reinforcing particles. Using this method, a mesoscale model of MICP-cemented sand is constructed that reflects the realistic features of the precipitated structure. On this basis, uniaxial tension, uniaxial compression, and direct shear loadings are applied to the model to systematically investigate the deformation and failure behavior under different stress states. To validate the proposed model, MICP-cemented sand columns are prepared and subjected to corresponding tension, compression, and shear tests, with the fracture process captured by high-speed imaging. The applicability and limitations of the model are assessed by comparing the simulated and experimental stress–strain responses and crack patterns. This framework is intended for a mechanism-oriented analysis of mesoscale fracture in MICP-cemented sand.

## 2. Methods

### 2.1. Establishment of the Numerical Model for MICP-Cemented Sand

#### 2.1.1. Materials and Constitutive Model

After MICP treatment, the precipitates form cementing bridges at grain contacts and within pore spaces. Consequently, the sand skeleton gradually evolves from a frictional granular assembly into a cemented porous medium, and load transfer between grains becomes increasingly governed by CaCO_3_ bonds rather than by purely geometric and frictional contacts [2,3,4]. This transition motivates the representation of MICP-cemented sand as a particle-reinforced composite consisting of an equivalent cementing matrix and cohesive interfaces [39].

In the present mesoscale model, a two-dimensional plane-stress finite element formulation is adopted. The MICP-cemented sand is idealized as a multiphase composite comprising sand grains, crystalline CaCO_3_ particles, and an amorphous MICP matrix [40]. The crystalline particles include calcite, aragonite, and vaterite, which are explicitly represented as elastic reinforcement phases, while the amorphous MICP phase is treated as a continuous matrix [39].

Following common practice in mesoscale models of concrete and asphalt mixtures containing random aggregates [41,42,43], pores and locally under-deposited regions below the selected mesoscale are not explicitly discretized. Instead, their homogenized mechanical effects are incorporated into the effective constitutive properties of the amorphous matrix. The matrix, therefore, represents the combined effects of amorphous calcium carbonate, bacterial residues, organic macromolecules, and submicron pores. The influence of these components is reflected in the effective elastic modulus, strength, and fracture energy of the matrix.

Sand grains and crystalline CaCO_3_ particles are treated as linear elastic solids, whereas damage and fracture are allowed within the amorphous matrix and along the matrix-particle interfaces [44,45]. These damage and debonding processes are described using a cohesive zone model (CZM) [46]. This idealization is supported by the fracture-surface SEM observations presented in Section 3.3, which show that most sand grains remain intact after failure, whereas cracking occurs mainly within the MICP precipitates and along the precipitate-sand interfaces. Therefore, under the low- or zero-confinement and monotonic loading conditions considered in this study, matrix cracking and interfacial debonding are regarded as the dominant failure mechanisms. It should be emphasized that the linear-elastic assumption applies only to the intact bulk response before cohesive damage initiation; the subsequent nonlinear response is governed by progressive cohesive damage within the MICP matrix and along matrix–particle interfaces.

The elastic properties of the sand grains and crystalline CaCO_3_ phases were taken from published experimental data [17,47,48,49,50,51,52], as summarized in Table 1. The adopted values include the elastic moduli, Poisson’s ratios, and densities of calcite, aragonite, vaterite, and sand grains. The amorphous MICP phase is also assumed to be linear elastic in its bulk state. Because its effective mechanical properties cannot be uniquely determined from the literature, their initial ranges were selected with reference to representative data for amorphous CaCO_3_ and CaCO_3_-organic composite phases [53,54,55].

A bilinear CZM was employed to describe cracking within the amorphous matrix and debonding along the interfaces between the amorphous phase and the crystalline CaCO_3_ particles or sand grains [45,46]. The normal and tangential initial stiffnesses of the cohesive interfaces were estimated from the elastic moduli of the adjacent materials and the element size using commonly adopted empirical relations [56]. Initial ranges for the cohesive strengths and fracture energies were constrained using data from MICP-related interface shear and pull-out tests and studies of sand-cement interface fracture [44,57,58]. The three loading conditions were used jointly to constrain a single parameter set. The tensile, shear, and compressive stress-strain curves mainly informed the corresponding normal, tangential, and compressive CZM parameters, respectively. Parameter selection considered the peak-strength level, overall curve shape, and post-peak response holistically, rather than requiring an exact match to a single experimental quantity.

Once the parameters were calibrated, all bulk material properties, CZM parameters, and boundary conditions were kept unchanged. Models A, B, and C differed only in their randomly generated particle arrangements. Model A was used during the calibration stage, whereas Models B and C were compared with the second and third experimental curves, which were not involved in parameter selection. Because the numerical particle distributions were not direct reconstructions of the actual specimen microstructures, these comparisons were intended to assess the out-of-sample consistency of the model in terms of response range, characteristic curve shape, and dominant failure mode, rather than to establish a one-to-one correspondence with individual specimens.

The material properties and CZM parameters adopted in this study are summarized in Table 1 and Table 2. The effective properties of the amorphous MICP phase and the interfaces depend strongly on the bacterial strain, calcium source, pH, grout composition, and curing conditions [21,59]. Therefore, the values listed in Table 1 and Table 2 should be regarded as equivalent parameters specific to the mixture and preparation procedure considered in this study, rather than as universal constants for all MICP systems. Applying this framework to other materials or treatment conditions would require corresponding experimental calibration before conducting quantitative analysis or prediction.

#### 2.1.2. Construction of the Geometric Model by a Multiphase Random Particle Generation and Placement Algorithm

**Figure 1:**
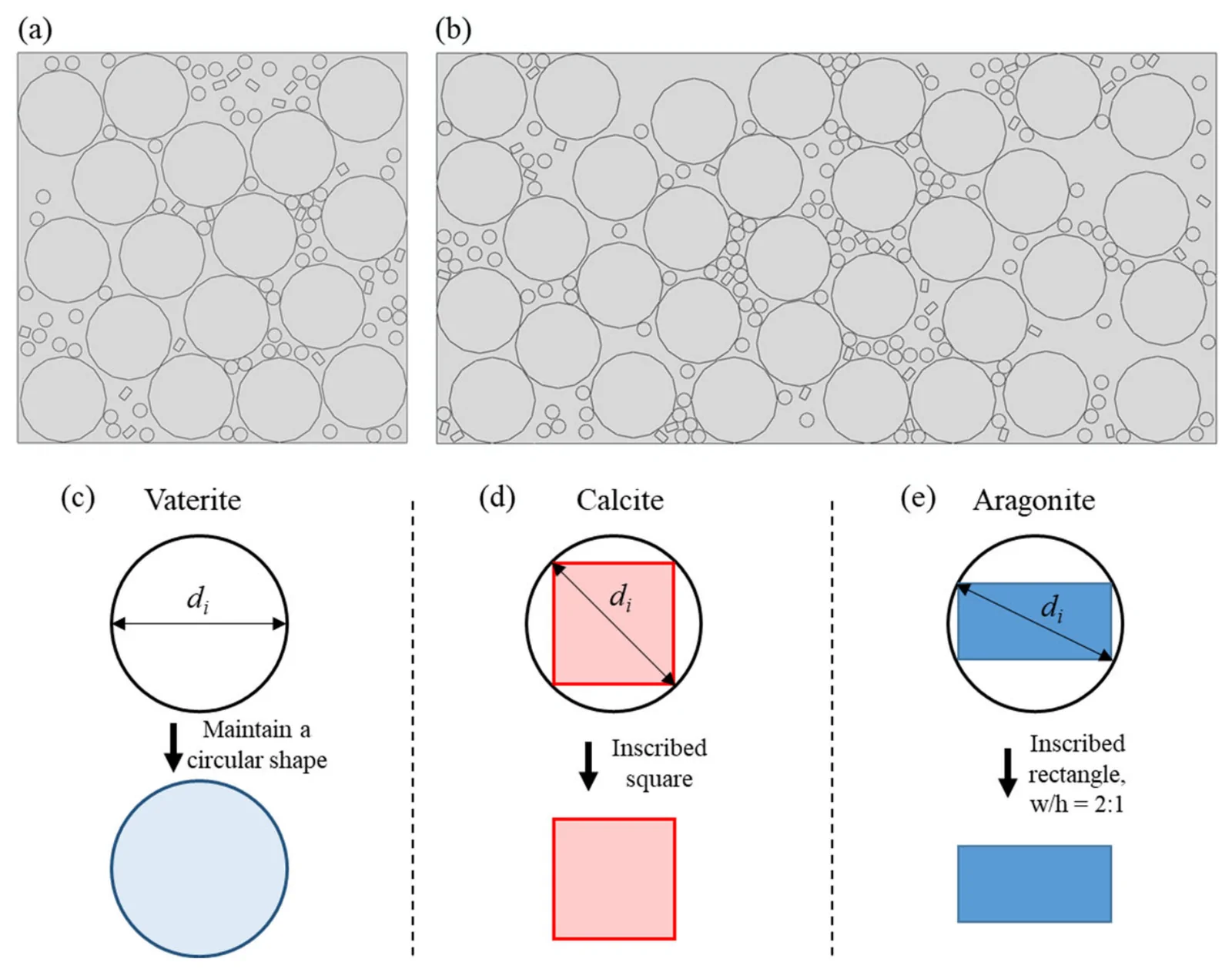
Geometric models and idealized particle-shape representation of MICP-cemented sand: (**a**) 100 μm × 100 μm model; (**b**) 200 μm × 100 μm model; (**c**) vaterite represented by a circle; (**d**) calcite represented by a square inscribed in the initially generated circle; and (**e**) aragonite represented by a rectangle with an aspect ratio of 2:1 inscribed in the initially generated circle.

To construct a two-dimensional geometric model of multiphase MICP-cemented sand, a multiphase random particle generation and placement algorithm is developed by extending the classical “two-phase circular particle placement algorithm” originally proposed for concrete mesoscale modeling [60,61]. The key extensions include: (1) introducing shape-correction factors to represent circular, square, and rectangular particles corresponding to sand grains and the different CaCO_3_ polymorphs (calcite, vaterite, and aragonite); (2) enforcing prescribed phase-specific volume fractions and particle size ratios consistent with experimental measurements; and (3) generating a non-overlapping DEM-type multiphase packing in PFC2D that can be directly imported into a CZM-based FE mesh. The core concept is that circular particles are first generated and placed in a non-overlapping manner, and then these circles are inscribed to obtain sand grains and different CaCO_3_ polymorph particles, as illustrated in Figure 1. This procedure can be implemented on the PFC2D discrete element platform. The detailed steps are as follows:

First, the number of particles for sand and different calcium carbonate crystal phases must be determined. In MICP-cemented sand, calcite crystals are typically blocky, aragonite crystals are prismatic, while sand grains and vaterite crystals are approximately spherical. Therefore, in the 2D mesoscale model of MICP-cemented sand, calcite grains are represented by squares, aragonite grains by rectangles with an aspect ratio of 2:1, and vaterite grains and sand grains by circles. Previous studies [26,27,62] have shown that the sand grain size in MICP-cemented sand is usually 5–10 times larger than the CaCO_3_ crystal size. In this study, the sand grain size is taken as 22 μm and the CaCO_3_ crystal size as 3.6 μm, giving a size ratio of 6.11. Experimental data for MICP-cemented sand columns [63,64,65] indicate that the sand volume fraction is typically about 65%, while the CaCO_3_ crystal volume fraction ranges from 4% to 20%. Under the experimental conditions of this study, the measured CaCO_3_ volume fraction of the MICP-cemented sand columns falls within 6.8–12.4%, within which the material no longer behaves as a purely granular medium but rather as a cemented porous medium. Since the precipitated CaCO_3_ includes not only crystalline phases but also amorphous calcium carbonate, the total volume fraction of CaCO_3_ crystals in the model is conservatively taken near the lower bound of this range, i.e., 7.5%.

Based on the volume fractions of sand and the different CaCO_3_ polymorphs (approximated as area fractions in 2D), the target placement area of each phase in the representative volume element (RVE) is first determined, and then the number of particles of each phase is obtained using the equivalent particle size and a shape correction factor. Let $A_{dom}$ denote the RVE area. According to the experimental and literature results mentioned above, the volume fractions in the plane approximation are taken as $\phi_{s}=0.65$ for sand and $\phi_{CaCO_{3}}=0.075$ for the total CaCO_3_ crystals. The target placement areas of sand and CaCO_3_ crystals are then:

(1) $$A_{s}^{target}=\phi_{s}A_{dom}$$

(2) $$A_{CaCO_{3}}^{target}=\phi_{CaCO_{3}}A_{dom}$$

To distinguish different polymorphs, a phase fraction coefficient $r_{i}$ is introduced (satisfying $\sum r_{i}=1$), where i∈{Va,Cc,Ar} denotes vaterite, calcite, and aragonite, respectively. The target placement area of each polymorph is then:

(3) $$A_{i}=r_{i}\cdot A_{CaCO_{3}}^{target}$$

To account for the effective area differences caused by different particle shapes, a shape correction factor $k_{i}$ is introduced and defined as the ratio of the actual effective particle area to the area of its circumscribed circle. Let $d_{i}$ denote the diameter of the circumscribed circle (equivalent diameter), whose area is $\pi d_{i}^{2}/4$. The effective area of a single particle of phase i can be written uniformly as:

(4) $$a_{i}=k_{i}\cdot \frac{\pi d_{i}^{2}}{4}\;a_{S}=k_{S}\cdot \frac{\pi d_{S}^{2}}{4}$$

where the shape correction factors $k_{i}$ for each phase are:

(5) $$\mathrm{Sand},\;\mathrm{vaterite}\;(\mathrm{circular}):\;k_{S}=k_{Va}=1$$

(6) $$\mathrm{Calcite}\;(\mathrm{square}):\;k_{Cc}=2/\pi$$

(7) $$\mathrm{Aragonite}\;(\mathrm{rectangle}):\;k_{Ar}=4\gamma /[\pi (\gamma^{2}+1)]$$

where γ=w/h is the aspect ratio of the rectangle. The equivalent diameter of sand grains is taken as $d_{S}=22\;\mu m$, and the equivalent diameter of CaCO_3_ crystals is taken as $d_{C}=3.6\;\mu m$ (i.e., $d_{i}=d_{C}$ for Va, Cc and Ar). Once the target area $A_{i}$ of each phase and the effective area $a_{i}$ of a single particle are determined, the required number of particles of each phase is given by:

(8) $$N_{i}=\left\lceil A_{i}/a_{i}\right\rceil \;\;N_{S}=\left\lceil A_{s}^{target}/a_{S}\right\rceil$$

where $\left\lceil \cdot \right\rceil$ denotes the ceiling function, which rounds the calculated particle number up to the nearest integer so that the total placed area is not smaller than the prescribed target area.

Second, the sand particles and the grains of various calcium carbonate crystal phases are generated and dispersed. Sand grains and CaCO_3_ particles are generated and placed in the domain under two constraints: (1) particle centers are uniformly and randomly distributed within the prescribed domain; (2) no newly generated particle is allowed to overlap with any previously placed particle. Before generating circular particles, the rectangular model domain is defined by the coordinates of its two opposite corners, ($x_{l},y_{l}$) and ($x_{r},y_{r}$). Under the assumption of statistically homogeneous mesoscale heterogeneity, the center $P_{n}(x_{n},y_{n})$ of each particle is sampled from a uniform distribution, with probability density functions:

(9) $$f_{X}(x)=\left\{\begin{array}{c} \frac{1}{x_{r}-x_{l}},\;\;\;\;\;\;x\in \left[x_{l},x_{r}\right],\\ 0,\;\;\;\;\;\;\;\;\;otherwise, \end{array}\right.$$

(10) $$f_{Y}(y)=\left\{\begin{array}{c} \frac{1}{y_{r}-y_{l}},\;\;\;\;\;\;y\in [y_{l},y_{r}]\\ 0,\;\;\;\;\;\;\;\;\;otherwise, \end{array}\right.$$

To check for overlap between any two circular particles with centers and radii ($x_{1},y_{1},r_{1}$) and ($x_{2},y_{2},r_{2}$), the following criterion is used:

(11) $$\sqrt{{\left(x_{1}-x_{2}\right)}^{2}+{\left(y_{1}-y_{2}\right)}^{2}}\geq r_{1}+r_{2}$$

If this inequality is satisfied, the two circular particles do not overlap.

After the initial placement, a brief dynamic relaxation step was applied to eliminate minor numerical penetrations caused by coordinate rounding and to achieve a stable particle configuration. A sufficiently high contact stiffness was assigned to both the particles and the model boundaries. Consequently, any slight particle-particle or particle-wall penetration generated a repulsive force, driving the particles to adjust until a stable and geometrically admissible packing was reached.

Third, the initially generated circular particles are geometrically transformed into specific shapes to represent different CaCO_3_ polymorphs. For calcite, a square is inscribed in the corresponding circle. For aragonite, a rectangle with an aspect ratio of approximately 2:1 is inscribed in the corresponding circle. Vaterite particles and sand grains retain their circular shapes. The particle data generated in PFC2D are exported and imported into ANSYS 2023 R2, where an APDL subroutine performs the corresponding geometric transformation, as illustrated in Figure 1c–e In this way, a multiphase CaCO_3_ particle assembly with realistic geometric shapes is constructed.

At this point, the mesoscale heterogeneous geometric model of the MICP-cemented sand is fully established, as shown in Figure 1. The proposed modeling and generation procedure can be readily extended from two-phase to three- or four-phase systems: once the volume fraction, particle size distribution, and shape factor of each phase are specified, the algorithm can automatically determine the phase proportions and perform geometric placement, thereby accurately controlling polymorph ratios while incorporating the effects of inscribed particle morphology.

#### 2.1.3. CZM Criterion and Establishment of the Finite Element Model

In the present model, the MICP-cemented sand is idealized as a particle-reinforced composite: sand grains together with calcite, aragonite, and vaterite crystals are treated as the reinforcing phases, while the amorphous phase-composed of amorphous calcium carbonate (ACC), bacterial residues, and organic macromolecules-serves as the matrix. The interfaces between the matrix and reinforcing particles are mechanically weak and tend to fail under loading. The cohesive zone model (CZM) is therefore used to describe damage initiation and crack propagation within the amorphous matrix and along matrix–particle interfaces [66]. In the present implementation, no single macroscopic crack path is prescribed. Cohesive elements are distributed within the amorphous MICP matrix and along matrix–particle interfaces, forming a network of potential fracture paths. The actual crack path develops through the progressive damage and failure of cohesive elements that satisfy the local traction–separation criterion and is therefore controlled by the local stress field and mesoscale heterogeneity. In the CZM framework, the bulk material remains elastic, while damage is represented by virtual cohesive interfaces. A bilinear traction–separation law is adopted [67]. The basic concept of the bilinear CZM is that the interface response is initially linear elastic, with stiffness governed by the prescribed normal and tangential initial stiffnesses. When the traction reaches a specified peak strength, damage initiates; thereafter, the traction degrades linearly with increasing separation until it drops to zero, corresponding to complete interface failure. The area under the traction-separation curve represents the fracture energy, which characterizes the energy dissipated during interface fracture. Therefore, crack propagation is governed not only by the local traction state but also by the fracture energy associated with cohesive separation. Regions with lower cohesive resistance require less energy for complete separation and are consequently more likely to develop into preferential fracture paths. By assigning distinct peak strengths and fracture energies in the normal and tangential directions and adopting an appropriate mixed-mode fracture criterion, the model can simultaneously capture interface opening (Mode I) and sliding (Mode II) fracture behavior. On this basis, a bilinear CZM is employed in this study to describe damage and fracture within the amorphous matrix and along the interfaces between the amorphous phase and the various particle phases, thereby enabling mesoscale simulation of the interface-dominated failure behavior of MICP-cemented sand under different loading conditions.

In the MICP-cemented sand column tests conducted by Zhao et al. [68], the volume fractions of vaterite, aragonite, and calcite in the precipitated CaCO_3_ particles were approximately 87.8%, 10.6% and 1.6%, respectively. In the present model, the prescribed area fractions were 65.0% for sand grains and 7.5% for the total crystalline CaCO_3_ phase. Accordingly, the nominal area fractions of vaterite, aragonite, and calcite were 6.585%, 0.795%, and 0.120%, respectively, while the remaining 27.5% of the domain was assigned to the equivalent amorphous MICP matrix. The same prescribed phase fractions were used in all numerical cases of this research. These cases differ only in the randomly generated spatial arrangement of the particles.

**Figure 2:**
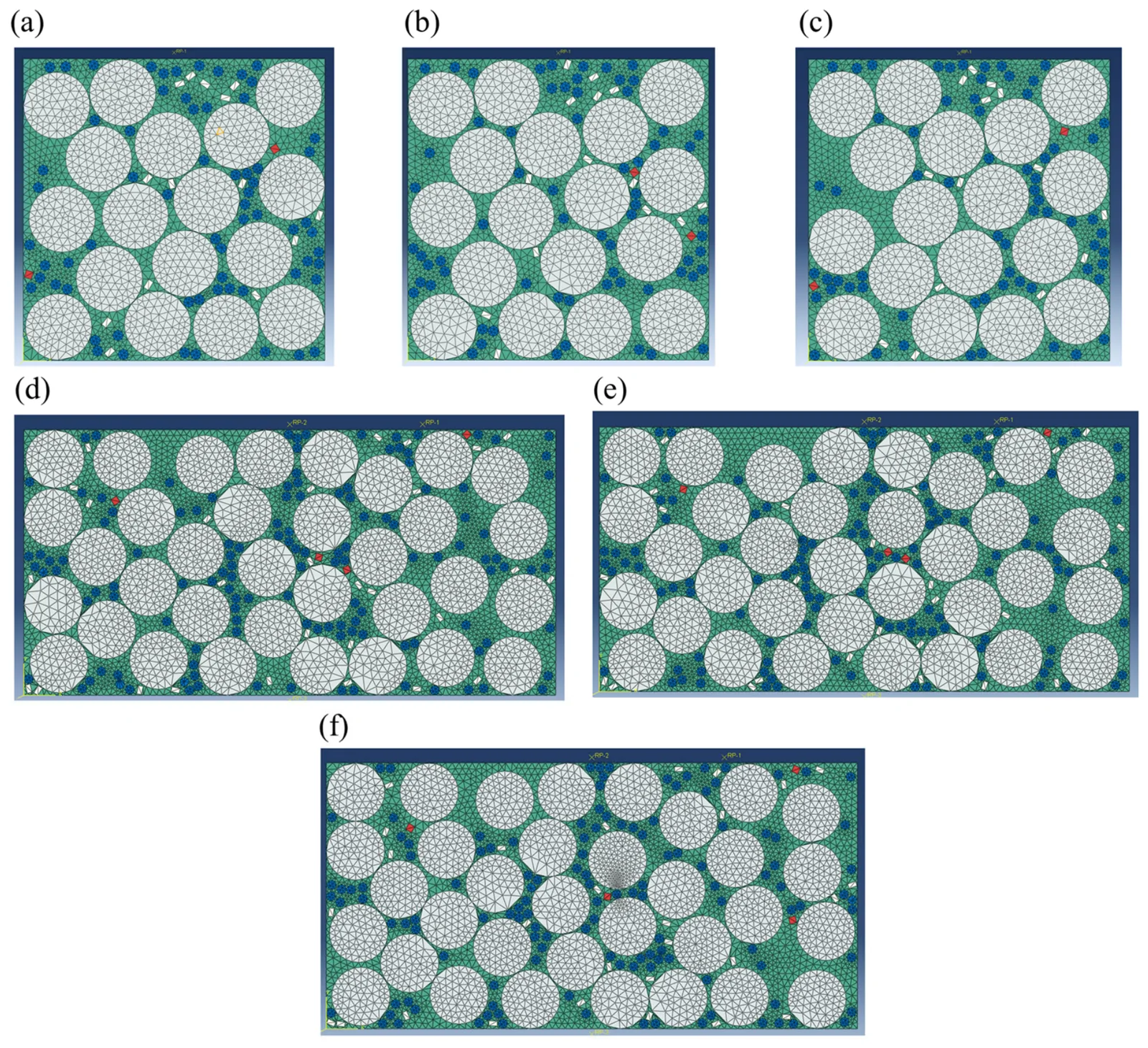
Finite element models of MICP-cemented sand with different sand-particle distributions: (**a**–**c**) models of size 100 μm × 100 μm; (**d**–**f**) models of size 200 μm × 100 μm.

To perform numerical simulations of uniaxial tension/compression and direct shear, two types of two-dimensional MICP-cemented sand models with overall sizes of 100 μm × 100 μm and 200 μm × 100 μm were generated using the proposed multiphase random particle generation and placement algorithm, as shown in Figure 1a,b. To investigate the influence of sand-particle distribution on the mechanical behavior of the cemented sand, each of the two model sizes was generated three times with different random seeds, resulting in three MICP-cemented sand finite element models with distinct sand-grain configurations. Under the plane stress assumption, the domains were discretized using solid triangular elements. Cohesive elements governed by a bilinear traction-separation CZM were inserted within the amorphous CaCO_3_ matrix and along the interfaces between the amorphous phase and the crystalline particles, as illustrated in Figure 2 [41].

**Figure 3:**
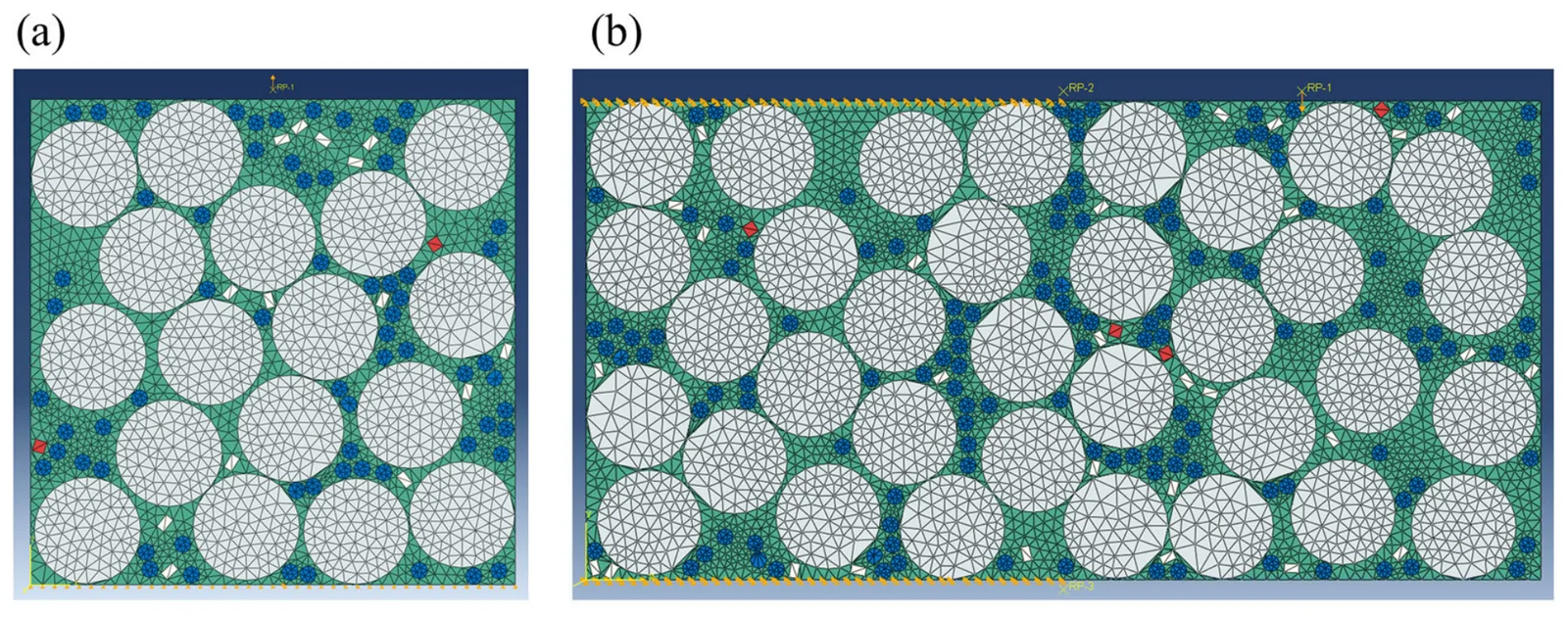
Finite element models of MICP-cemented sand: (**a**) 100 μm × 100 μm; (**b**) 200 μm × 100 μm.

The boundary conditions are set as follows: to perform uniaxial tension and compression simulations, the bottom edge of the 100 μm × 100 μm MICP-cemented sand model is constrained in the vertical direction, and the horizontal displacement at the bottom center is also fixed. A prescribed vertical displacement of 0.06 μm is then applied at the top edge in the upward or downward direction to generate tensile or compressive loading, as shown in Figure 3a. For direct shear simulations, the top and bottom edges of the left half of the 200 μm × 100 μm model are constrained in both the horizontal and vertical directions, while a vertical downward displacement of 0.06 μm is applied to the top edge of the right half, as illustrated in Figure 3b. To mitigate the mesh dependence of the numerical results [60], a series of trial analyses with different mesh sizes were conducted. It was found that an element size of 2.5 μm provides an appropriate balance between computational accuracy and efficiency.

The stress–strain responses were obtained from the force–displacement data extracted from the finite element simulations. An out-of-plane thickness of 1 μm was assumed for all two-dimensional models. The nominal stress was calculated by dividing the resultant reaction force by the nominal cross-sectional area of the model, and the nominal strain was calculated by dividing the prescribed displacement by the initial model height:

(12) $$\sigma =\frac{F}{bt},\;\varepsilon =\frac{∆}{H}$$

where F is the resultant reaction force, b is the model width, t=1 μm is the assumed model thickness, ε is the prescribed displacement, and H is the initial model height. For direct shear, the same normalization procedure was used, with the shear reaction force and applied shear displacement used to obtain the nominal shear stress and shear strain. This procedure was applied consistently to the simulated stress-strain curves mentioned later.

#### 2.1.4. CZM Parameter Sensitivity and Model Robustness

To evaluate the sensitivity of the model to uncertainties in the CZM parameters, all cohesive parameters assigned to the MICP matrix and matrix–particle interfaces were uniformly scaled relative to their baseline values. These parameters include the initial stiffness, tensile strength, tensile fracture energy, compressive strength, compressive fracture energy, shear strength, and shear fracture energy. The scaled parameter was defined as

(13) $$P_{\lambda }=\lambda P_{0}$$

where $P_{0}$ is the baseline value listed in Table 2 and λ is the scaling factor. Three values, λ=0.9, 1.0 and 1.1, were considered. Thus, λ=0.9 means that each CZM parameter was reduced to 90% of its baseline value.

**Figure 4:**
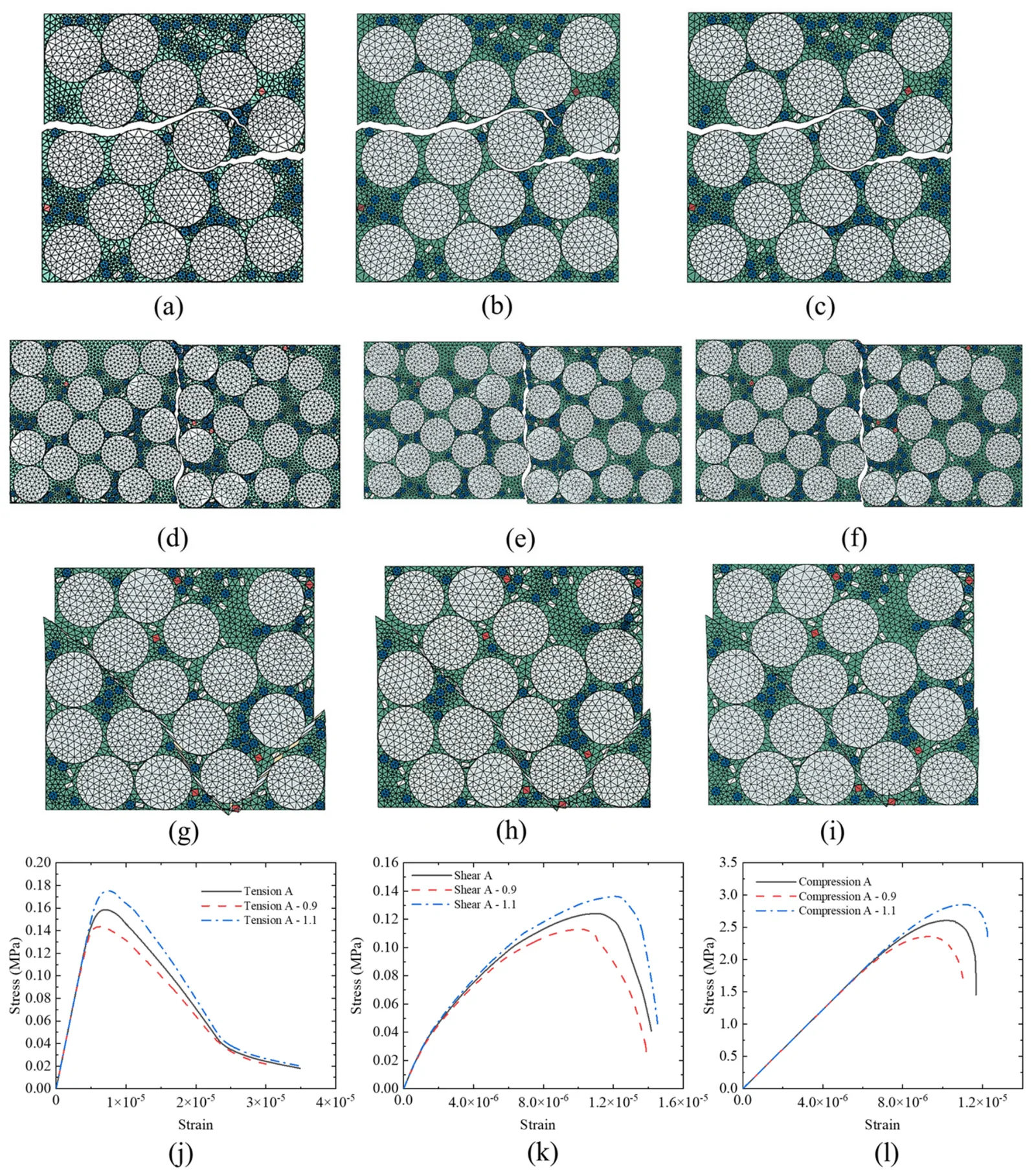
Crack paths and stress–strain responses of MICP-cemented sand under different CZM parameter scaling factors: (**a**–**c**) tensile models with λ = 1.0, 0.9 and 1.1, respectively; (**d**–**f**) direct shear models with λ = 1.0, 0.9 and 1.1, respectively; (**g**–**i**) uniaxial compression models with λ = 1.0, 0.9 and 1.1, respectively; (**j**–**l**) stress–strain curves under tension, direct shear and uniaxial compression, respectively. Here, λ denotes the uniform scaling factor applied to the CZM parameters of the MICP matrix and matrix-particle interfaces.

The particle distribution, mesh, and boundary conditions were kept unchanged. The resulting stress–strain curves and final crack paths under tension, shear, and compression are compared in Figure 4.

As shown in Figure 4j–l, the initial ascending branches are nearly identical under all three loading conditions, indicating that a ±10% variation in the CZM parameters has little influence on the initial elastic response. Differences become more evident after damage initiation. Reducing the parameters to 0.9 times the baseline values lowers the peak stresses and leads to earlier softening and failure. The lower peak of Tension A-0.9 is caused by the reduced cohesive resistance. With lower cohesive strength and fracture resistance, damage initiates at a lower applied load and propagates more rapidly, resulting in an earlier loss of load-carrying capacity and a lower tensile peak. Increasing them to 1.1 times the baseline values raises the peak stresses and corresponding peak strains, while improving the post-peak load-carrying capacity. These results show that the CZM parameters mainly affect damage initiation, peak strength, and post-peak softening, but have limited influence on the initial stiffness.

The main crack paths remain largely consistent across all parameter sets. In Figure 4a–c, the tensile cracks are approximately perpendicular to the loading direction and propagate mainly through the matrix and along matrix-particle interfaces while bypassing sand grains. In Figure 4d–f, the shear cracks remain concentrated within the prescribed shear region and develop into through-going shear bands. In Figure 4g–i, the compression cracks form inclined shear bands with local deflection. Although the parameter changes affect crack opening, local deflection, and branching, they do not significantly alter the location, overall direction, or dominant failure mode of the main cracks.

Overall, varying the CZM parameters by ±10% changes the predicted peak strength and post-peak response but does not alter the dominant crack paths or interface-controlled failure mechanisms under tension, shear, and compression. This indicates that the main conclusions regarding crack evolution and failure modes are not merely artifacts of a specific calibrated parameter set but remain reasonably robust within the investigated parameter range.

#### 2.1.5. Assumptions and Applicability of the Modeling

The present study employs a 2D plane-stress finite element model to represent a typical mesoscale section of MICP-cemented sand. The model focuses on the effects of multiphase composition and particle distribution on the stress–strain response, crack paths, and failure modes. Under monotonic uniaxial tension, direct shear, and unconfined compression, the specimens generally develop a dominant crack band or shear band whose overall direction can be identified from a representative section. Therefore, the 2D model retains the essential mesoscale features at a relatively low computational cost and can effectively describe crack initiation, deflection, coalescence, and propagation.

Sand grains and crystalline CaCO_3_ particles are treated as linear elastic phases, while damage and fracture are allowed within the amorphous MICP matrix and along the matrix-particle interfaces. This assumption is supported by the higher stiffness of the sand grains and crystalline particles relative to the weak cementing phase and its interfaces. Section 3.3 also demonstrate that most sand grains remain intact after failure, whereas cracks mainly propagate through the MICP deposits and along the precipitate-sand interfaces. Thus, under the low- or zero-confinement and monotonic loading conditions considered here, matrix cracking and interfacial debonding are regarded as the dominant failure mechanisms. Local brittle fracture of crystalline precipitates is not modeled separately, and its average effect is represented indirectly through the effective properties of the matrix and interfaces.

These assumptions introduce several limitations. First, the 2D model cannot fully represent 3D grain coordination, out-of-plane cementing bridges, or spatial load-bearing networks. This may affect the predicted stiffness, peak strength, and local stress distribution. In real specimens, cracks may deflect, branch, merge, or bypass particles through the specimen thickness, whereas cracks in the present model are restricted to the modeling plane. Therefore, the predicted crack-initiation sites, crack length, tortuosity, and energy dissipation may differ from those in real 3D specimens.

Second, the model does not explicitly account for particle sliding, rolling, interlocking, or crushing, nor for frictional contact and fragment reloading after interface failure. Consequently, it cannot reproduce stiffness degradation caused by particle crushing under high stress or high confinement, and it may underestimate the residual load-carrying capacity during the post-peak stages of shear and compression. These mechanisms become increasingly important at large deformation, when particle rearrangement, frictional interlocking, and recontact may dominate the mechanical response.

In addition, the actual MICP deposits are spatially nonuniform because of solution transport, bacterial attachment, local pore-throat geometry, and grain-contact conditions. Local pores, weakly cemented regions, and variations in deposit thickness are not explicitly reconstructed in the present model. Their average mechanical effects are instead incorporated into the effective elastic modulus, strength, and fracture energy of the amorphous MICP matrix. This simplification may affect the local crack-initiation site, crack branching pattern, crack tortuosity, and scatter in peak strength. Therefore, the model is not intended to precisely predict the local crack path or peak strength of an individual specimen.

Overall, the model is mainly applicable to weakly to moderately cemented materials under low or zero confinement and monotonic quasi-static loading, where matrix cracking and interfacial debonding dominate failure. It can be used to analyze the initial response, dominant crack direction, main failure modes, and relative differences among mesoscale structures. However, it is not intended for high confinement, large deformation, significant particle crushing, strong frictional rearrangement, cyclic loading, or impact loading, nor for the precise prediction of 3D crack morphology, post-peak residual strength, or absolute fracture-energy dissipation. Within this range of applicability, the model reasonably captures the transverse tensile fracture, direct-shear band development, and inclined compressive shear failure observed in the experiments.

### 2.2. MICP-Cemented Sand Under Tensile, Shear, and Compressive Loading

#### 2.2.1. Materials

**Figure 5:**
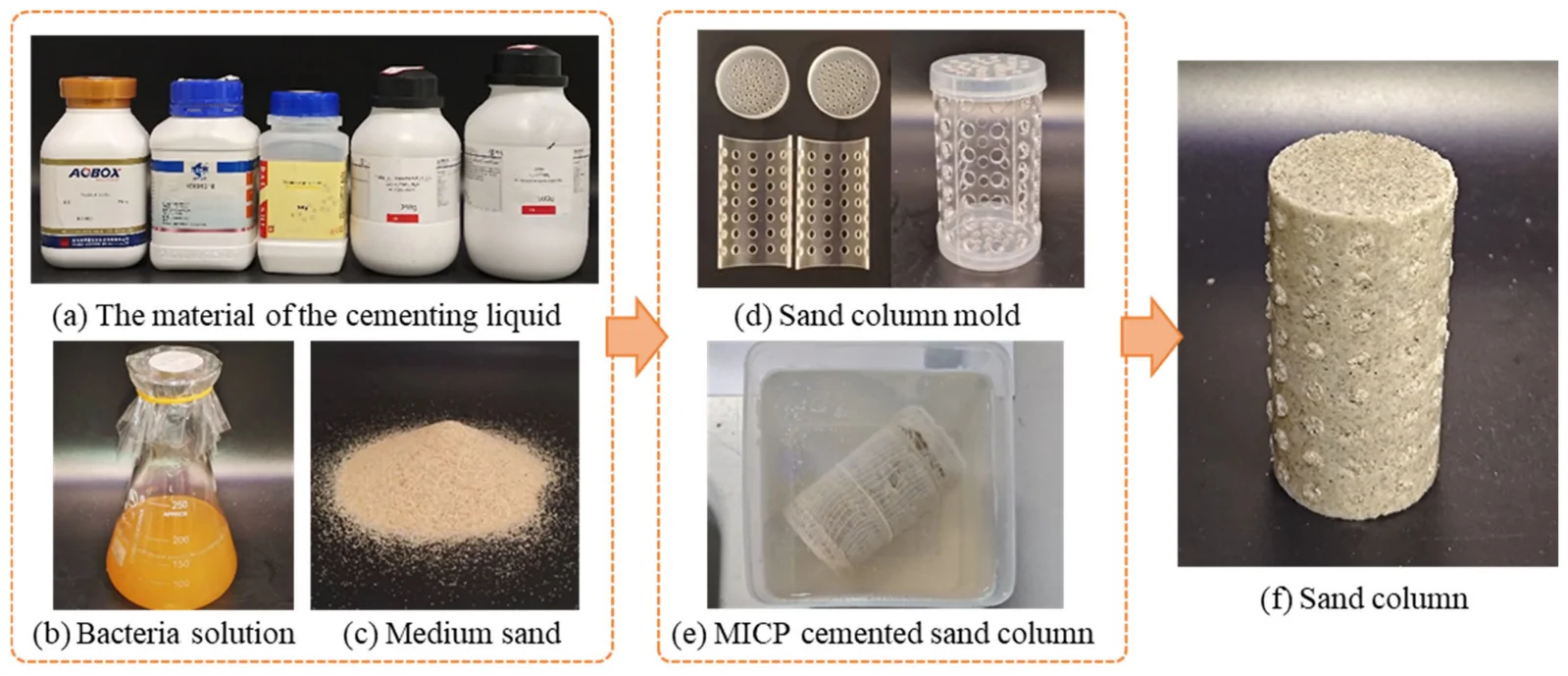
Preparation procedure for MICP-cemented sand columns.

The bacterium used in this study was Sporosarcina pasteurii (ATCC 11859, American Type Culture Collection), obtained from the China General Microbiological Culture Collection Center (Shanghai, China). The bacterial suspension was cultured in the laboratory following the procedure below: (1) The strain was inoculated into a liquid growth medium containing, per liter, 20 g yeast extract, 10 g NH_4_Cl, 10 mg MnSO_4_·H_2_O, and 24 mg NiCl_2_·6H_2_O, and the pH was adjusted to 8.5 using 1 M NaOH; (2) The culture was incubated in a shaking incubator at 30 °C and 200 rpm for approximately 18–24 h; (3) When the optical density of the culture at 600 nm (OD_600_) reached 1.5–2.0, the bacterial suspension was considered ready for MICP treatment, as shown in Figure 5b.

Yeast extract and MnSO_4_·H_2_O were supplied by Macklin Biochemical Co., Ltd. (Shanghai, China). NH_4_Cl, NiCl_2_·6H_2_O, and NaOH were obtained from Sinopharm Group (Shanghai, China). Calcium acetate and urea were purchased from Xilong Scientific Co., Ltd. (Foshan, China), nutrient broth from Beijing Aoboxing Bio-tech Co., Ltd. (Beijing, China), and NaHCO_3_ from Tianjin Baishi Chemical Co., Ltd. (Tianjin, China). The sand used for preparing the MICP-cemented sand columns was locally sourced river sand from Foshan, China, sieved to a particle size range of 0.35–0.50 mm, as shown in Figure 5c.

#### 2.2.2. Preparation of MICP-Cemented Sand Columns

The cementation solution for the MICP reaction was prepared by dissolving 0.5 M urea and 0.5 M Ca(CH_3_COO)_2_, with 3 g/L nutrient broth as a carbon and energy source, and 10 g/L NH_4_Cl and 2.12 g/L NaHCO_3_ added to buffer the pH. The materials used are shown in Figure 5a.

The MICP-cemented sand columns were prepared using an immersion method as follows. (1) Specimen preparation: Dried and sieved sand was placed into transparent acrylic cylindrical molds. The molds were gently tapped to compact the sand column and approximate a naturally deposited state, as shown in Figure 5d. (2) Bacterial inoculation: The sand columns were fully immersed in the bacterial suspension for 3.5 h to ensure thorough penetration of the pores and sufficient attachment of cells to grain surfaces and pore walls. The columns were then removed and left to stand at room temperature for 0.5 h to promote bacterial adsorption and fixation. (3) Precipitation: The inoculated sand columns were subsequently transferred into the cementation solution and completely immersed, as illustrated in Figure 5e. The MICP reaction was carried out at 30 °C, with each reaction cycle lasting approximately 20 h. During this period, bacteria hydrolyzed urea to produce CO_3_^2−^, which combined with Ca^2+^ in solution to precipitate CaCO_3_ and form bonds between sand grains. To increase the amount of cementation, three precipitation cycles were performed in total. After each immersion, the columns were removed and gently rinsed to remove free ions from the surface, and then re-immersed in fresh cementation solution. (4) Curing and drying: After completing all precipitation cycles, the sand columns were kept at 30 °C for 24 h to allow further CaCO_3_ crystal growth and interface bonding. Finally, the specimens were dried in a vacuum oven at 60 °C for 12 h to obtain the final samples for mechanical testing, as shown in Figure 5f.

The experimental program was designed as a controlled numerical–experimental comparison rather than a parametric optimization study. Therefore, the MICP treatment conditions were kept fixed, while the loading mode was varied among tension, direct shear, and compression. Three replicate specimens were tested under each loading condition. The repeated tests were used to examine the consistency of the dominant failure mode within each loading condition. Because local MICP heterogeneity can also affect the detailed crack path, the experiments are used here to provide representative mechanical responses and qualitative fracture patterns rather than to establish loading mode as the exclusive factor controlling fracture morphology.

#### 2.2.3. Tensile, Compressive and Shear Tests and Observation of the Failure Process

To characterize the mechanical behavior and failure evolution of MICP-cemented sand columns under different loading conditions, uniaxial tension, uniaxial compression, and direct shear tests were conducted, and the entire failure process was recorded using a high-speed imaging system. The test setups and measurement procedures are described below.

(1) Uniaxial tensile test

**Figure 6:**
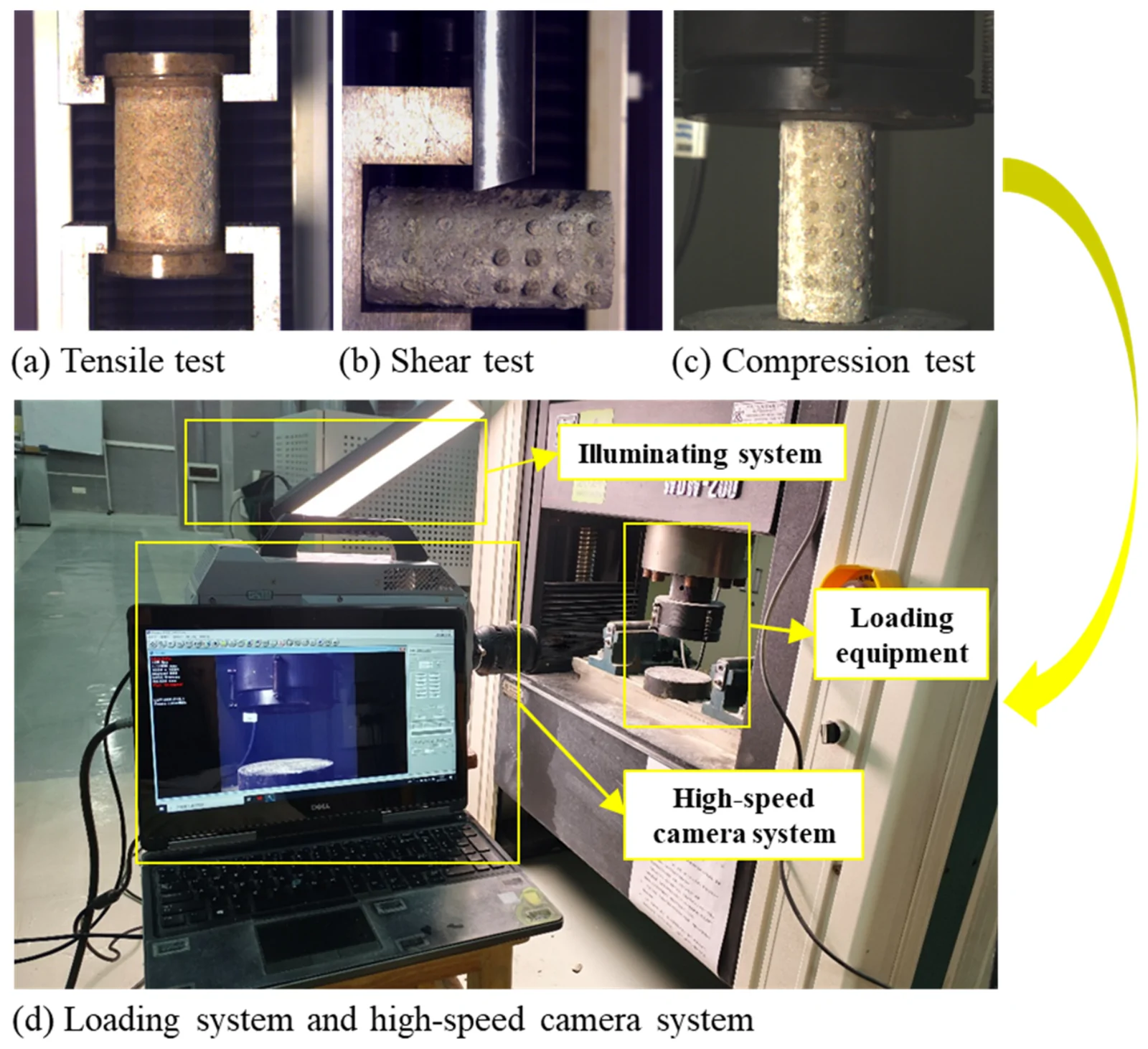
Setups for tensile, compressive and shear tests, and observation of the failure process.

The tensile behavior of MICP-cemented sand columns was examined using a custom-made axial tension fixture, as shown in Figure 6a. Both ends of each specimen were wrapped with a thin epoxy layer to form an I-shaped profile, and the fixture gripped the epoxy-wrapped ends so that the load could be transferred uniformly along the specimen axis. The tests were performed on an electronic universal testing machine (BLD1026, Bolaide, Dongguan, China) under displacement control at a loading rate of 2 mm/min. During testing, load and crosshead displacement were recorded in real time and converted into tensile stress–strain curves. Each test was continued until complete failure of the specimen.

(2) Direct shear test

Shear behavior was evaluated using a direct shear fixture. As shown in Figure 6b, the fixture consists of a clamping unit on the left and a loading rod on the right, with the tip of the loading rod machined flat. The clamping unit was kept fixed, while the loading rod applied a vertical displacement under displacement control at a rate of 2 mm/min, inducing shear failure in the central part of the specimen. The system continuously recorded shear force and displacement, from which shear stress–strain curves were obtained. The tests were carried out until the specimens were fully sheared through.

(3) Uniaxial compression test

Uniaxial compression tests were conducted on an electronic universal testing machine (WDW-200, Kexin, Changchun, China), using steel platens to apply axial compressive load to the top and bottom ends of the specimens, as shown in Figure 6c. The loading was displacement-controlled at a rate of 2 mm/min. Prior to testing, the specimen ends were ground flat to reduce eccentric loading and minimize end-friction effects on the results. Load and displacement were recorded in real time and used to calculate compressive stress–strain curves. Each test was continued until the specimen was completely crushed or failed.

(4) High-speed imaging system

To capture the full fracture process, a high-speed camera system (FASTCAM SA-Z, Photron, San Diego, CA, USA) was employed for all three types of tests, as illustrated in Figure 6d. The camera settings were as follows: frame rate 500 fps; image resolution 520 × 520 pixels; and illumination provided by a high-intensity LED panel to ensure clear visualization of crack edges. The recorded images were post-processed using a threshold-based segmentation procedure to generate binary crack maps, which enabled clear identification of crack initiation and propagation in the brittle and quasi-brittle failure processes of the MICP-cemented sand columns. All experimental data and crack evolution observations were subsequently compared with the finite element simulation results to evaluate the reliability of the model in terms of strength prediction, failure mode reproduction, and consistency of crack paths.

#### 2.2.4. Determination of CaCO_3_ Content

To determine the actual CaCO_3_ content in the specimens, acid dissolution tests were performed on representative MICP-cemented sand columns. First, specimens after mechanical testing were dried in an oven at 60 °C to constant mass, and the dried mass $m_{0}$ was recorded. The specimens were then immersed in a dilute hydrochloric acid solution to fully dissolve the CaCO_3_. After the reaction was completed, the remaining sand grains were rinsed with distilled water and dried again to constant mass, and the residual mass $m_{1}$ was recorded. The mass fraction of CaCO_3_ can be calculated as:

(14) $$\omega_{CaCO3}=\frac{m_{0}-m_{1}}{m_{0}}$$

In total, five samples were taken from different positions along the MICP-cemented sand columns. The measured CaCO_3_ mass fractions ranged from 6.9% to 12.6%. Considering the typical densities of CaCO_3_ and sand as $\rho_{CaCO_{3}}=2.71\;g/{cm}^{3}$ and $\rho_{S}=2.65\;g/{cm}^{3}$, respectively, the corresponding volume fraction of CaCO_3_ can be obtained from the mass fraction as:

(15) $$\phi_{CaCO_{3}}=\frac{\omega_{CaCO3}/\rho_{CaCO_{3}}}{\omega_{{CaCO}_{3}}/\rho_{CaCO_{3}}+\left(1-\omega_{{CaCO}_{3}}\right)/\rho_{S}}$$

For the specimens in this study, the CaCO_3_ volume fraction is thus approximately 6.8–12.4%. This range is consistent with previously reported CaCO_3_ contents for MICP-cemented sands prepared under similar mixture proportions and treatment conditions, indicating that the overall cementation level of the specimens falls within a typical range for engineering applications.

## 3. Results and Discussion

### 3.1. Mechanical Response and Crack Evolution Behavior

#### 3.1.1. Comparison Between Numerical Simulation and Experimental Results Under Uniaxial Tension

**Figure 7:**
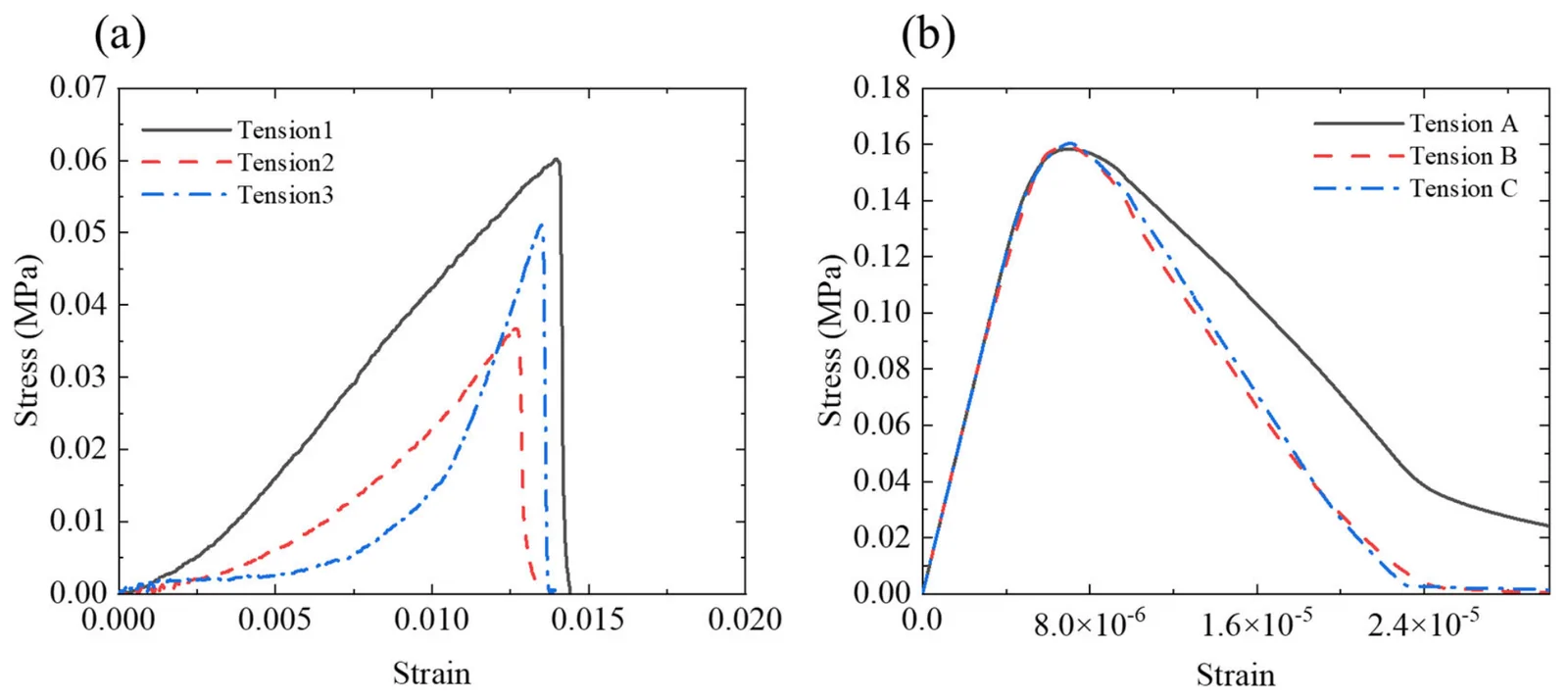
(**a**) Tensile stress–strain curves from experiments; (**b**) tensile stress–strain curves from the finite element models.

Figure 7 compares the experimental and numerical stress–strain curves of MICP-cemented sand columns under uniaxial tension. In terms of overall shape, both results exhibit a typical brittle fracture response. The three experimental curves (Figure 7a) are relatively steep and drop sharply after reaching the peak load. The three numerical curves (Figure 7b) all show an initial linear elastic stage, followed by a short non-linear softening segment, and then rapid post-peak instability.

However, noticeable differences arise between the experimental and numerical curves in strength level, strain scale, and post-peak softening behavior. First, the numerically predicted peak tensile strength (average ≈ 0.160 MPa) is significantly higher than that of the present test series (average ≈ 0.049 MPa), indicating that the model overestimates the tensile capacity of the tested specimens. Although the simulated strength falls within ranges reported in previous studies, this only indicates that its order of magnitude is reasonable and does not replace direct comparison with the present experiments. The discrepancy is mainly attributed to the use of uniform interface properties, the absence of large pores and continuous weakly cemented zones, the two-dimensional and scale simplifications, and the overly idealized random microstructure. The scatter in the experimental peak strengths is mainly attributed to the non-uniform MICP deposition and local weakly cemented regions, whereas the numerical models use uniform material and interface properties and therefore show much smaller variation in peak strength. This difference reflects the idealization of the current model rather than a numerical error. Therefore, the model is more suitable for analyzing interface-dominated tensile failure and crack paths than for accurately predicting the peak tensile strength of the tested specimens. Nevertheless, reported tensile strengths of MICP-cemented sands in the literature typically fall in the range of 0.1–0.3 MPa [63,68,69], and the numerical results of this study lie within this range. Second, the experimental peak strains are on the order of 10^−2^, whereas the numerical peak strains are on the order of 10^−5^, indicating a clear difference in strain magnitude. This discrepancy is mainly attributed to the way strain is measured in the tests: the experimental strain is obtained by dividing the crosshead displacement of the testing machine by the overall specimen height, and therefore includes not only the tensile deformation of the specimen itself, but also deformation and compliance from the grips, epoxy end caps, and other components (e.g., sensors), which amplifies the apparent global strain. Therefore, the composition of the simulated and experimental responses is different, and they cannot be compared. This is consistent in the shear and compression tests. Third, the numerical curves in Figure 7b exhibit a more extended post-peak softening branch, i.e., the specimens retain some load-carrying capacity after the peak, whereas the experimental curves drop almost instantaneously after the peak, indicating more abrupt failure. This difference implies that the CZM interface parameters in the model promote a relatively stable crack propagation process, while the specimens prepared by the immersion method exhibit pronounced heterogeneity, with numerous pores and weakly cemented zones. Once a propagating crack encounters such weak regions, it can rapidly cut through the section, leading to a steeper post-peak drop in the experimental curves. It is worth noting that the uniaxial tensile tests on MICP-cemented sand conducted by Nafisi et al. [64] also show tensile stress–strain curves with a pronounced post-peak softening tail, which is consistent with the numerical results in this study and further supports the credibility of the simulation.

**Figure 8:**
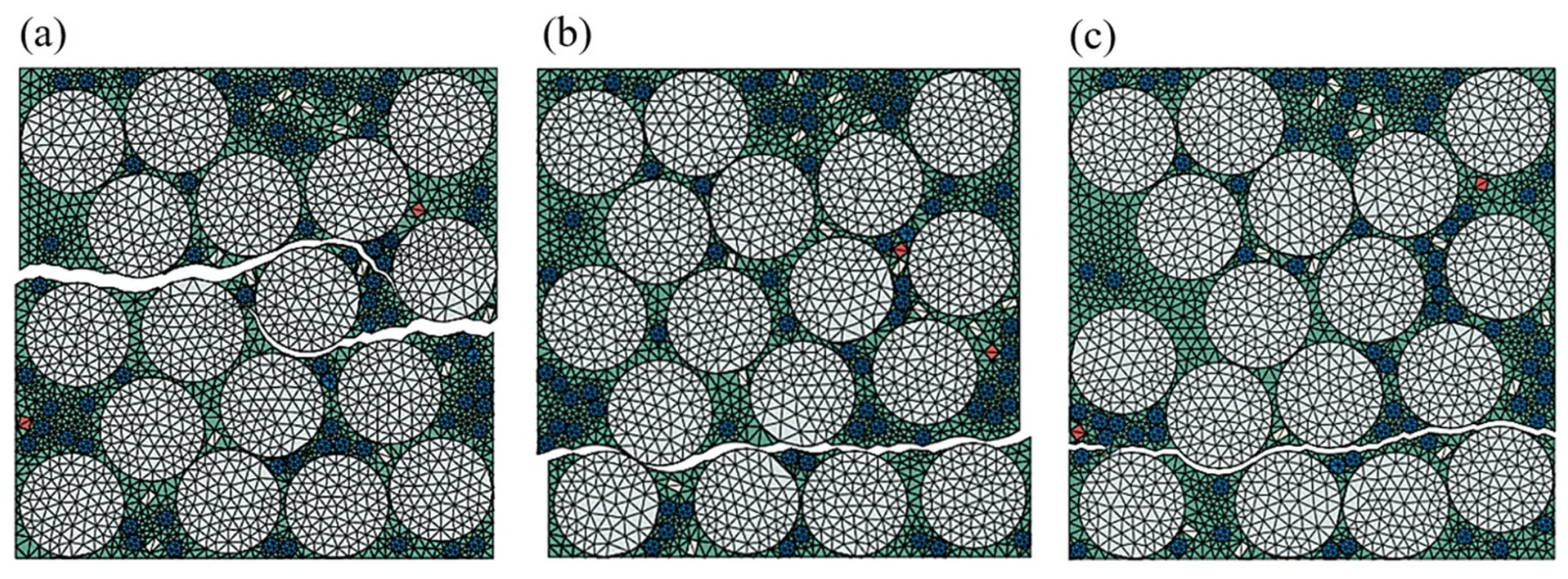
Crack paths after tensile failure for three models with different sand-particle distributions: (**a**) Tensile A; (**b**) Tensile B; (**c**) Tensile C.

Figure 8 shows the crack paths after tensile failure for the three models with different sand-particle distributions. For clarity, the deformation is magnified by a factor of 300. A comparison of the crack patterns in these three models leads to the following observations. First, in all cases, the cracks propagate approximately perpendicular to the loading direction, indicating that tensile failure of the cemented sand is governed by the maximum principal tensile stress. Second, crack initiation and propagation occur mainly along the interfaces between the CaCO_3_ precipitates and sand grains, as well as within the precipitates themselves. Since the interfaces are modeled using a CZM, once the damage variable reaches the critical value, the interface undergoes an opening-sliding failure; the cracks then bypass the sand grains along their boundaries rather than cutting through them, resulting in a typical interfacial debonding failure mode. Third, the sand particle distribution has a pronounced influence on the selection of crack paths. When propagating cracks are blocked by larger sand grains, the crack trajectories are forced to deflect, leading to more tortuous paths. In such cases, adjacent cracks must bypass the sand grains before coalescing, resulting in a longer and more gradual post-peak softening branch. This behavior corresponds to a longer post-peak descending branch in the stress–strain curve and improved tensile load-carrying capacity and crack resistance, as observed in the curve of model Tensile A (Figure 7b) and its crack pattern (Figure 8a). In contrast, when adjacent cracks link with little obstruction, rapid crack localization produces a steeper post-peak decline, as seen in models Tensile B and Tensile C (Figure 8b,c). These results indicate that the post-peak descending branch of the stress–strain curve is more sensitive to the microstructure and crack path, and that the scatter observed in the descending branch is mainly attributable to differences in crack propagation processes.

**Figure 9:**
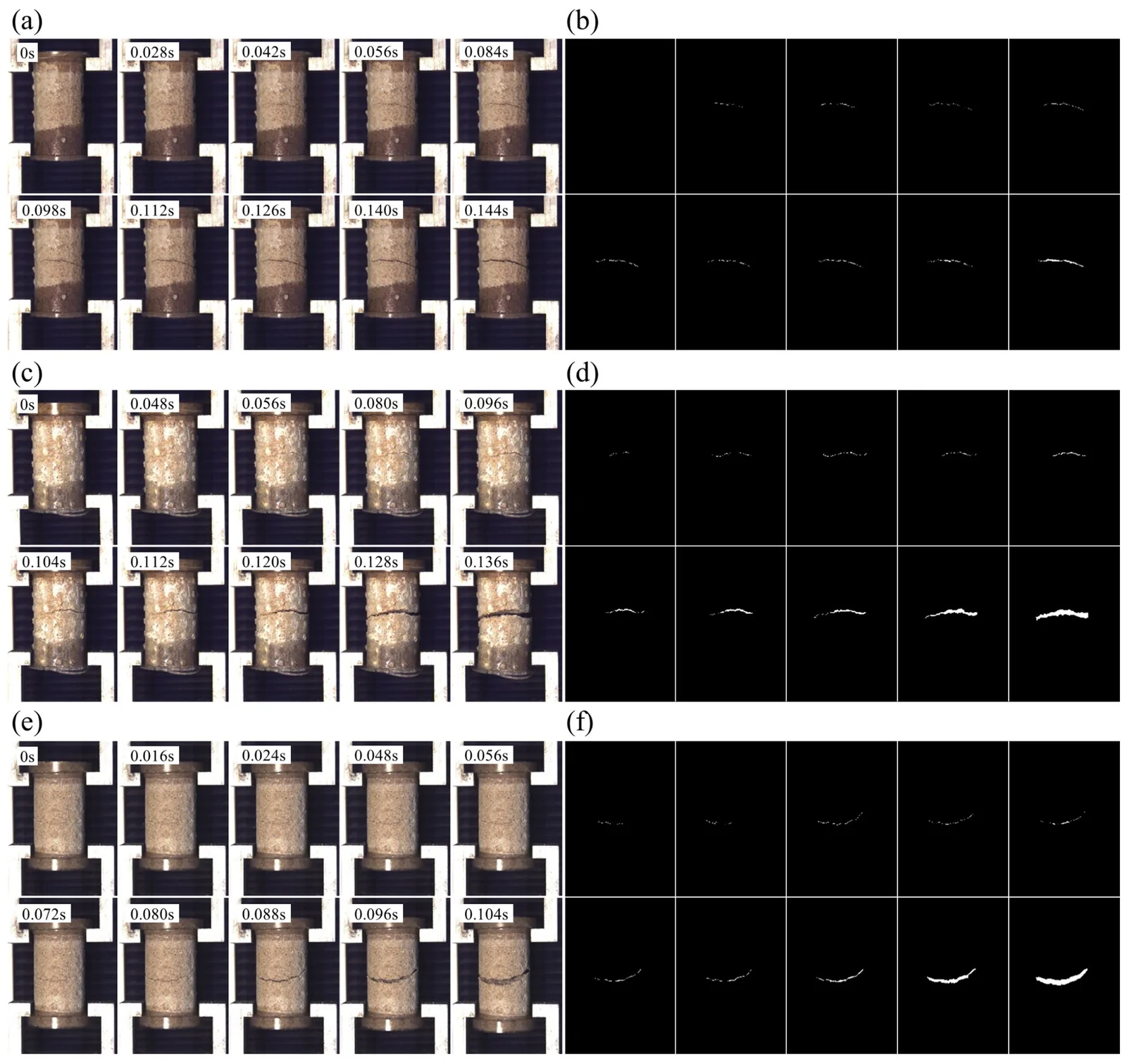
Crack evolution process and binarized crack identification for three MICP-cemented sand columns under tensile loading: (**a**,**b**) Tensile 1; (**c**,**d**) Tensile 2; (**e**,**f**) Tensile 3.

Figure 9a–f presents the crack evolution process and corresponding binarized crack maps for three MICP-cemented sand columns under uniaxial tension. Several observations can be made. First, in all specimens, the cracks propagate approximately perpendicular to the loading direction, which is consistent with the numerical results. Similar experimental findings were also reported by Nafisi et al. [64] and Zeng et al. [69], who observed that under uniaxial tension, the fracture planes in MICP-cemented sands are nearly orthogonal to the loading direction. Second, the crack paths exhibit a tortuous pattern rather than a perfectly straight through-crack, which agrees with the simulations. This tortuosity arises because the non-uniformity of MICP leads to spatial variations in interface strength; cracks tend to initiate in regions with thinner cementation layers or lower CaCO_3_ content, and then propagate along these weak zones. As loading progresses, neighboring cracks coalesce to form a zigzag crack band. This correspondence indicates that the interface strength settings in the CZM successfully capture the weak-interface effects induced by the heterogeneous MICP deposition observed in the experiments.

#### 3.1.2. Comparison Between Numerical Simulation and Experimental Results Under Direct Shear

**Figure 10:**
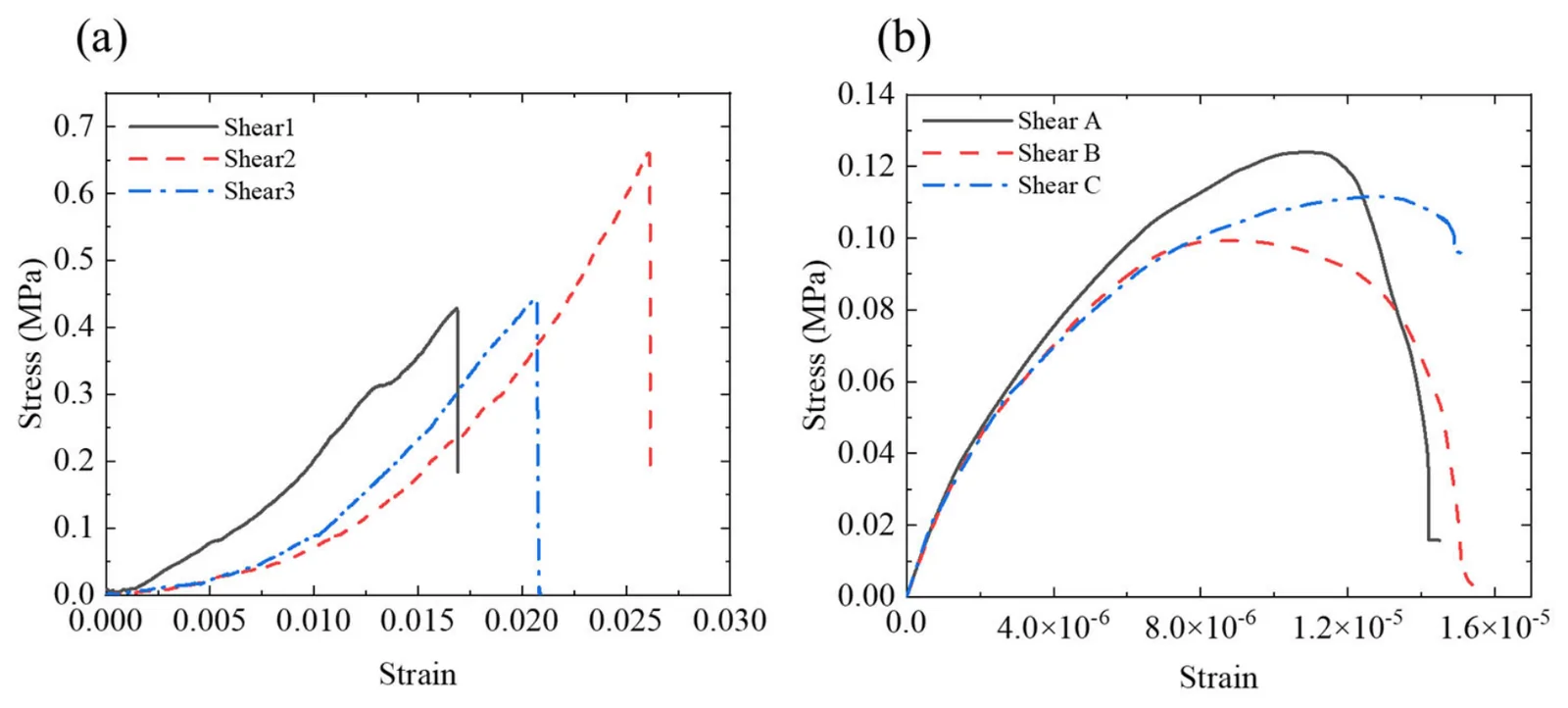
(**a**) Shear stress–strain curves from experiments; (**b**) shear stress–strain curves from the finite element models.

Figure 10 compares the experimental and numerical stress–strain curves of MICP-cemented sand columns under direct shear. In terms of overall shape, the three experimental curves (Figure 10a) exhibit a typical quasi-brittle response: the shear stress increases approximately linearly at first, followed by a rapid drop after reaching the peak. The three numerical curves (Figure 10b) also show a clear softening stage, with a relatively fast post-peak decay, consistent with a quasi-brittle interface-softening response. The ascending branches of the three simulated curves almost coincide, whereas their softening and descending segments progressively diverge. Overall, the numerical model reproduces the quasi-brittle shear behavior reasonably well.

Nevertheless, differences are observed between the experimental and numerical curves in terms of strength level, strain scale, and softening characteristics. First, the simulated peak shear stresses (average ≈ 0.115 MPa) fall within the range of shear strength values reported in previous MICP studies (0.02–0.8 MPa) [70,71,72,73], and the experimental peak stresses of this study (average ≈ 0.510 MPa) also lie within this interval. However, the current model still underestimates the macroscopic shear strength of the specimens. This discrepancy may arise from the simplified treatment of shear mechanisms. While the model employs the CZM to describe matrix cracking and matrix-particle debonding, it does not fully account for frictional sliding, surface roughness, particle interlocking, or the reloading of residual cementing bridges after interface failure. Furthermore, in the direct shear tests, fixture constraints may also introduce additional normal stresses near the shear zone, thereby increasing the apparent shear strength of the experimental specimens. The scatter in the experimental shear peaks has the same origin as discussed for the tensile tests, mainly reflecting the heterogeneous MICP deposition and local weakly cemented regions. Second, the experimental peak strains are on the order of 10^−2^, whereas the simulated peak strains are on the order of 10^−5^, which is similar to the discrepancy observed in tension. Third, the simulated curves are more idealized, exhibiting a nearly linear ascending branch, a smooth peak, and a relatively regular softening stage, while the experimental curves tend to drop more abruptly after the peak. This suggests that the chosen CZM interface parameters in the model promote relatively stable shear crack propagation, whereas the real specimens, prepared by immersion, contain numerous pores and weakly cemented zones; once a propagating shear crack interacts with these weak regions, rapid localization and sudden loss of load-carrying capacity can occur, resulting in a steeper post-peak drop in the experiments.

**Figure 11:**
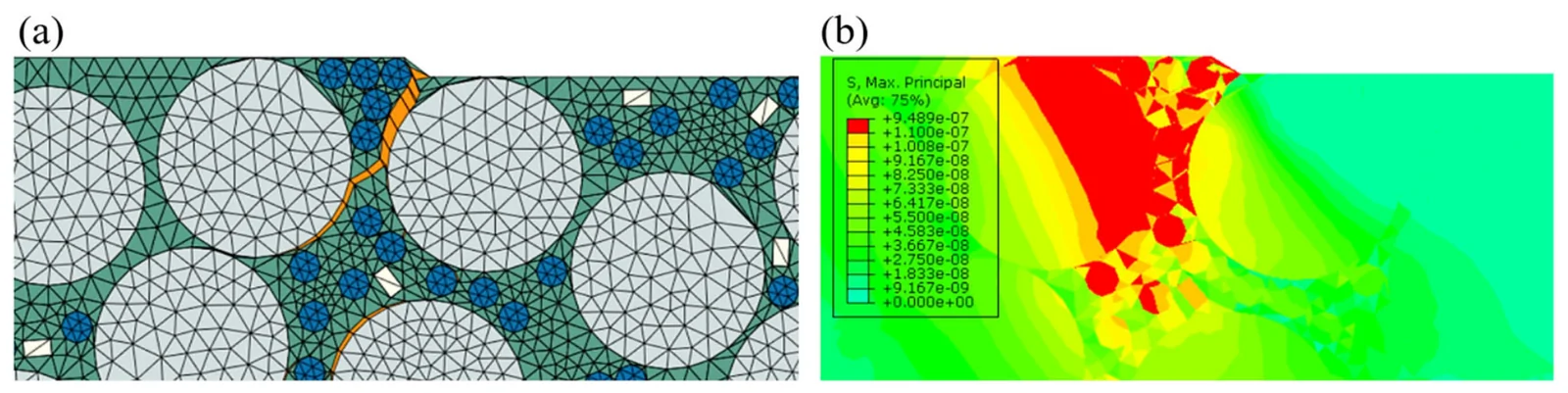
Shear model Shear C: (**a**) crack initiation; (**b**) first principal stress distribution(The stress values are shown in N/μm^2^ in the finite-element output, $1\;N/{\mu m}^{2}=10^{6}\;MPa$, e.g., “1e-7” means “0.1 MPa”).

Figure 11a shows the onset of cracking in the third shear model (Shear C). The crack-initiation state was identified from the damage evolution of the cohesive elements, and the first principal stress distribution was extracted at the same load step. Under direct shear loading, the crack initiates near the top of the shear plane and then propagates along a direction inclined at approximately 45° to the shear plane. This indicates that, in the early stages of crack initiation and propagation, crack growth is governed by the maximum principal tensile stress. A similar phenomenon was reported by Yin et al. [74] in their experimental and numerical investigation of direct shear cracking in asphalt mixtures. Figure 11b presents the distribution of the first principal stress at the moment of crack initiation in model Shear C, clearly showing that both the crack initiation site and the subsequent propagation path are located within regions of high first principal stress.

**Figure 12:**
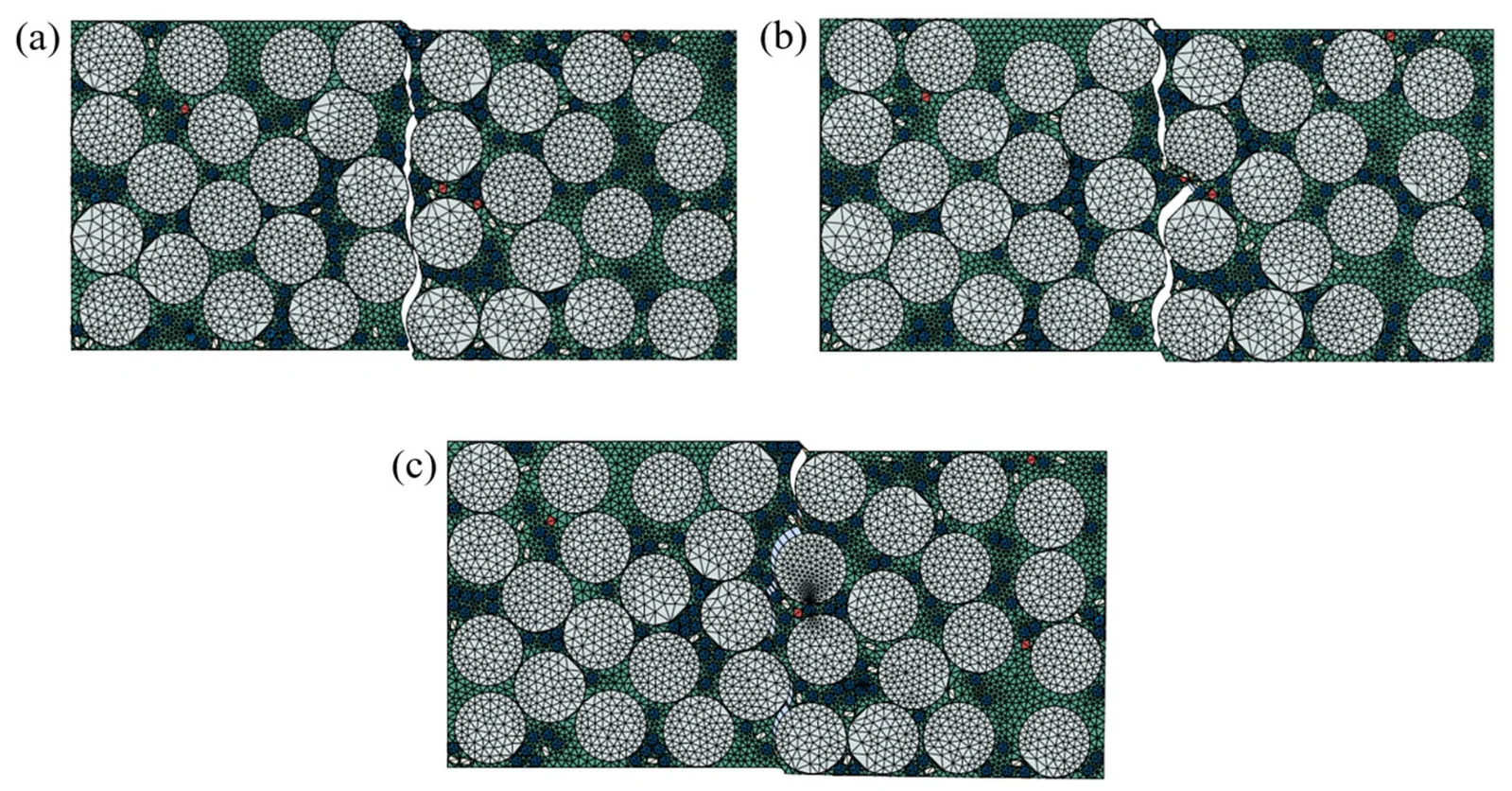
Crack paths after shear failure for three models with different sand-particle distributions: (**a**) Shear A; (**b**) Shear B; (**c**) Shear C.

Figure 12 shows the deformed configurations of three MICP-cemented sand models with different sand-particle distributions after direct shear failure. For clarity, the deformation is magnified by a factor of 2000. Several observations can be made. First, in all three models, the final fracture paths almost coincide with the shear plane. This indicates that, although crack initiation is initially governed by the maximum principal tensile stress, crack propagation during the main shear failure stage is controlled by the maximum shear stress, and the cracks continue to grow along the shear plane in a tortuous manner. Second, due to the packing of sand grains and the multiphase nature of the precipitates, the stress field in the vicinity of the shear plane is highly heterogeneous. Local stress concentrations promote crack deflection, giving rise to tortuous crack paths; in addition, the arrangement of sand grains influences crack propagation, such that when a propagating crack encounters a sand grain, it tends to bypass the grain rather than cutting through it. Third, crack initiation and propagation occur mainly along the interfaces between the CaCO_3_ precipitates and sand grains, as well as within the precipitates themselves, exhibiting a typical interfacial debonding failure mode.

**Figure 13:**
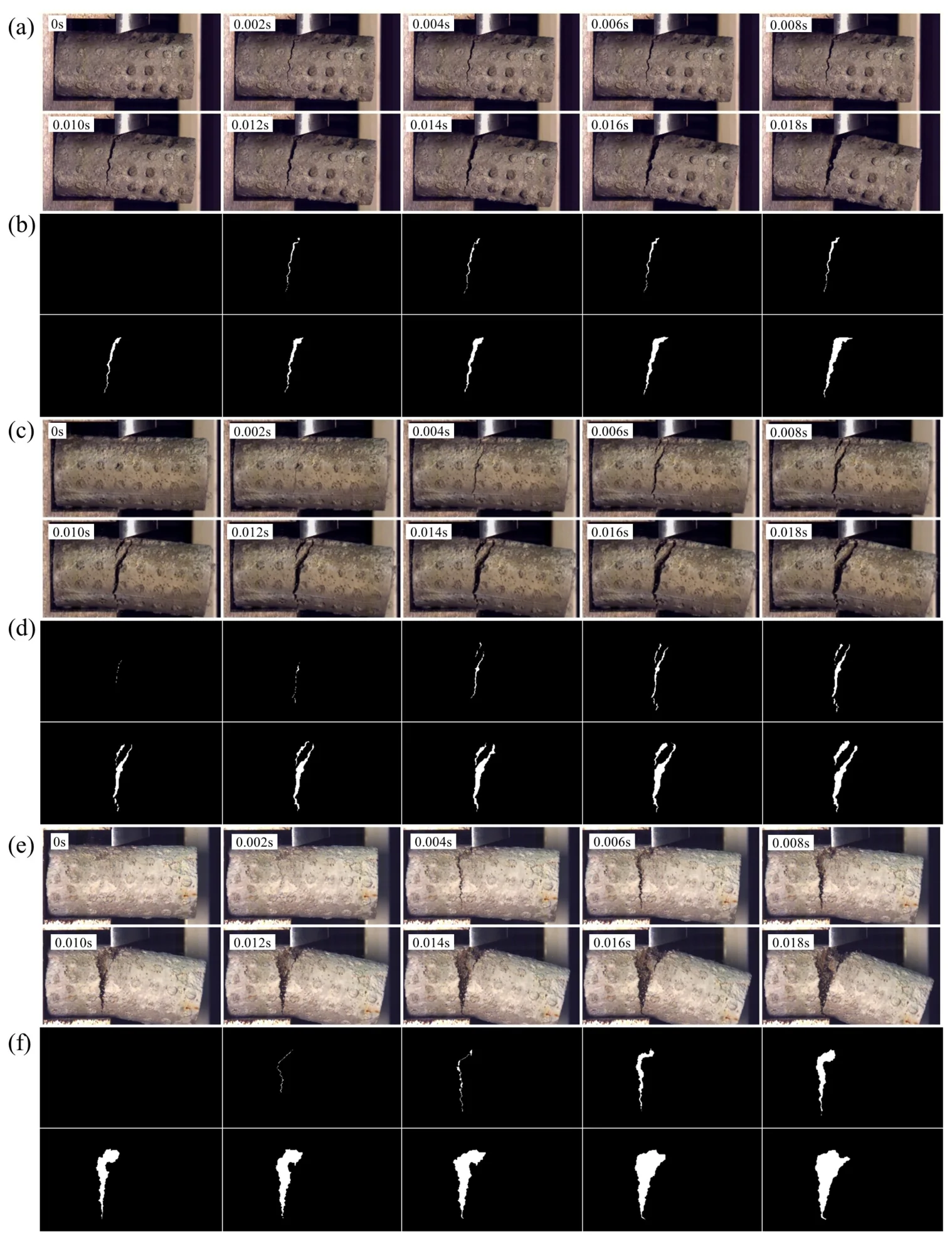
Crack evolution process and binarized crack identification for three MICP-cemented sand columns under shear loading: (**a**,**b**) Shear 1; (**c**,**d**) Shear 2; (**e**,**f**) Shear 3.

Figure 13a–f shows the crack evolution process and corresponding binarized crack maps for three MICP-cemented sand columns under direct shear loading. All three specimens exhibit crack development patterns that are consistent with the numerical results. First, cracks initiate in the vicinity of the shear plane and, with increasing shear displacement, progressively propagate along this plane to form a stable shear band. Second, the crack paths are tortuous and tend to bypass sand grains rather than cutting straight through them, eventually leading to a distinct single-sided or double-sided shear-slip failure. Similarly, Kariminia et al. [75] and Jia et al. [76] reported that, under direct shear loading, the fracture surface of MICP-cemented sand is parallel to the shear plane and that the sand grains on the fracture surface remain largely intact, indicating that cracks propagate mainly along the interfaces between sand grains and precipitates, as well as within the precipitated CaCO_3_. The experiments and simulations exhibit qualitative agreement in the dominant crack direction, shear band location, and final through-going crack pattern.

#### 3.1.3. Comparison Between Numerical Simulation and Experimental Results Under Uniaxial Compression

**Figure 14:**
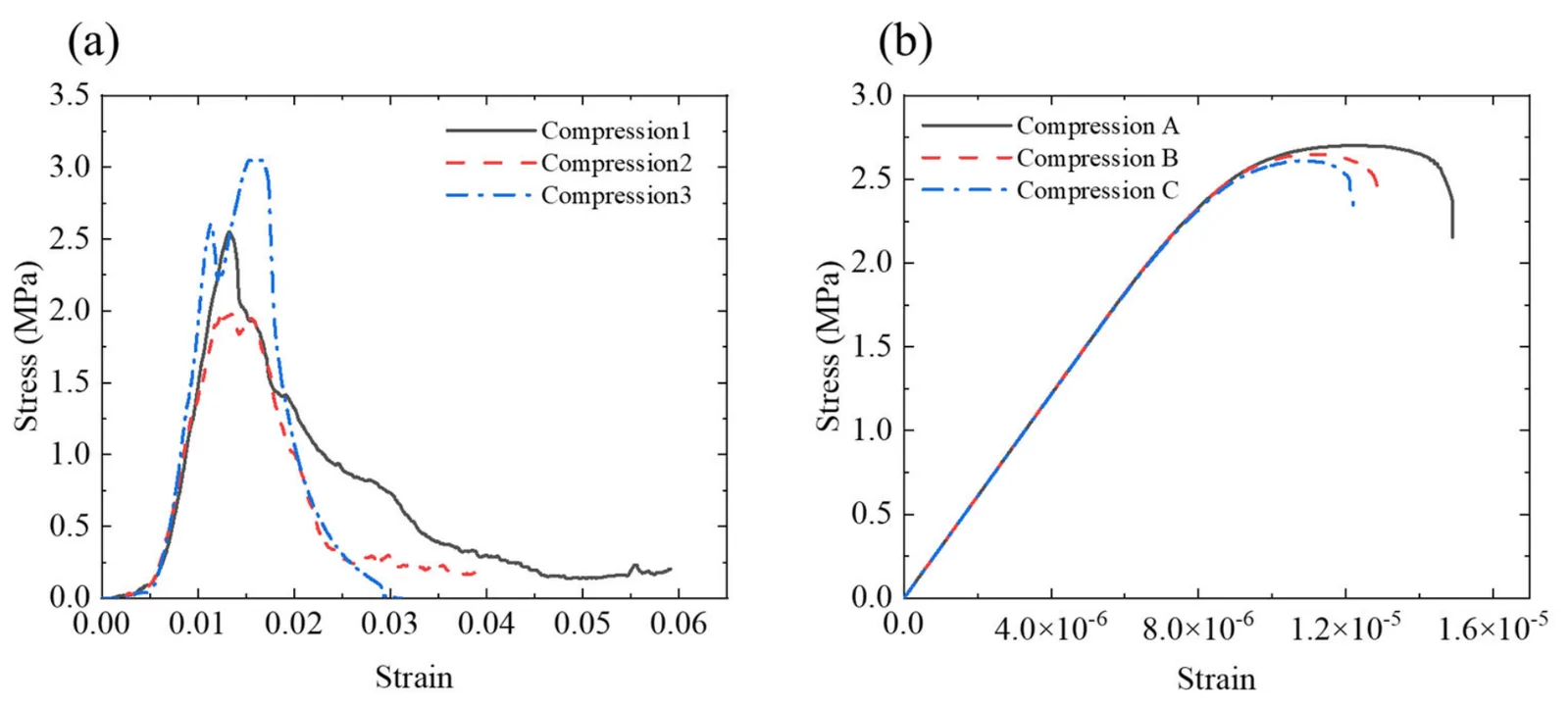
(**a**) Compressive stress–strain curves from experiments; (**b**) compressive stress–strain curves from the finite element models.

Figure 14 compares the experimental and numerical stress–strain curves of MICP-cemented sand columns under uniaxial compression. In terms of overall shape, the three experimental curves (Figure 14a) exhibit a typical brittle-frictional mixed failure behavior. The pre-peak ascending branches are very steep, indicating high equivalent stiffness of the specimens in the initial compression stage. Near the peak, some specimens show more than one local maximum (e.g., Compression 2 and Compression 3 in Figure 14a), implying that multiple shear bands may form during loading; local failures and subsequent load redistribution occur alternately, leading to fluctuations in the curve around the peak. After the peak, the stress drops rapidly and clear softening is observed. However, in contrast to the tensile and shear tests, the stress does not fall immediately to zero: a noticeable residual strength is maintained and then gradually decays, forming a relatively long tail. This indicates that, although cementation bridges progressively fail in the post-peak stage, the compressed, fragmented particles remain in contact, rearrange, and form new load-bearing structures, thus providing significant residual load-carrying capacity. The three numerical curves under uniaxial compression (Figure 14b) also display a typical quasi-brittle failure response. They show an almost ideal linear elastic ascending branch before reaching a distinct peak, followed by a post-peak descending segment. Compared with the experiments, the numerical curves exhibit a faster decay of the residual branch, reflecting the fact that mechanisms such as particle frictional interlocking and reloading of crushed fragments are not fully represented in the present model. The simulated peak stresses (average ≈ 2.65 MPa) are in good agreement with both the test results of this study (average ≈ 2.53 MPa) and previously reported compressive strengths of MICP-cemented sands (typically in the range of 1–3 MPa) [63,68,69]. The scatter in the experimental compressive peaks can be attributed to the same MICP heterogeneity discussed for the tensile tests. Overall, the numerical model reproduces the quasi-brittle behavior and peak strength level under uniaxial compression well, supporting the reliability of the simulation results.

**Figure 15:**
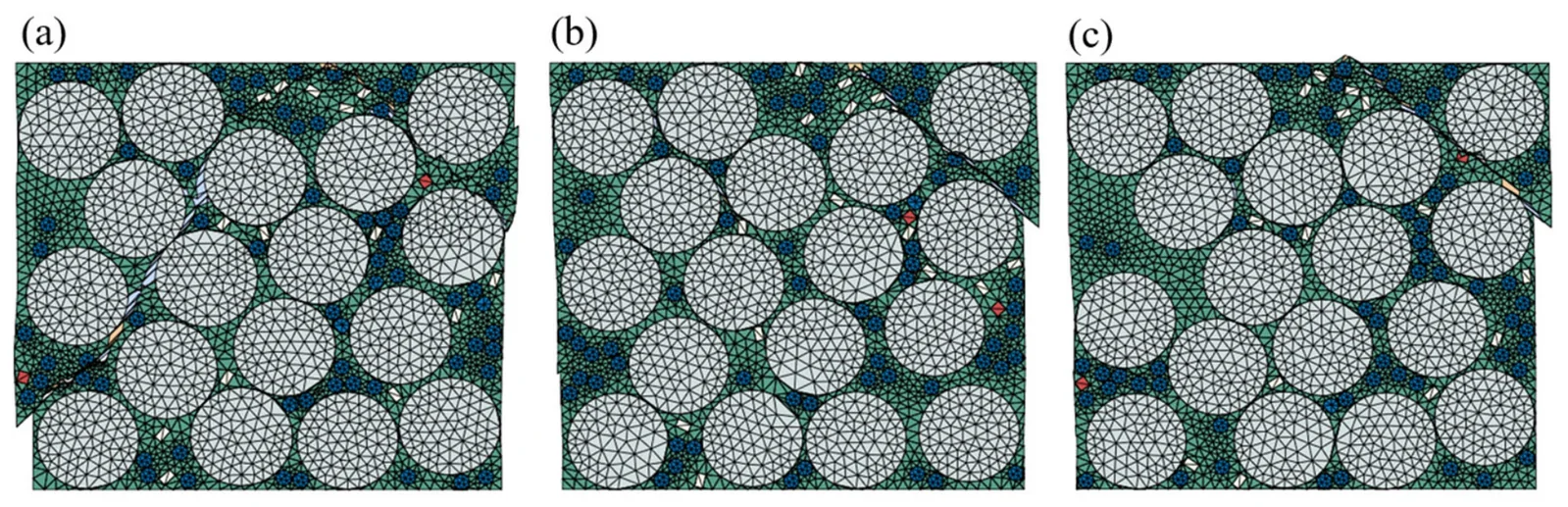
Crack paths after compressive failure for three models with different sand-particle distributions: (**a**) Compression A; (**b**) Compression B; (**c**) Compression C.

Figure 15 shows the deformed configurations of three models with different sand-particle distributions after compressive failure. For clarity, the deformation is magnified by a factor of 5000. In all three models, the final fracture paths are inclined at approximately 45° to the loading direction, indicating that the failure is governed by the maximum shear stress and can be classified as shear-dominated failure. Similarly, Nafisi et al. [64] and Yan et al. [77] reported that, under uniaxial compression, the fracture planes in MICP-cemented sand are inclined at about 45° to the loading direction. A comparison of the crack paths under different sand-particle distributions shows the following. First, regardless of the specific sand arrangement, cracking occurs primarily along the interfaces between the CaCO_3_ precipitates and sand grains, as well as within the precipitates themselves. Second, the sand particle distribution has a pronounced influence on the initiation sites and propagation paths of cracks. When a propagating crack is disturbed or blocked by larger sand grains, the crack path deviates locally and then returns to its original propagation direction after bypassing the obstacle, resulting in a tortuous fracture pattern.

**Figure 16:**
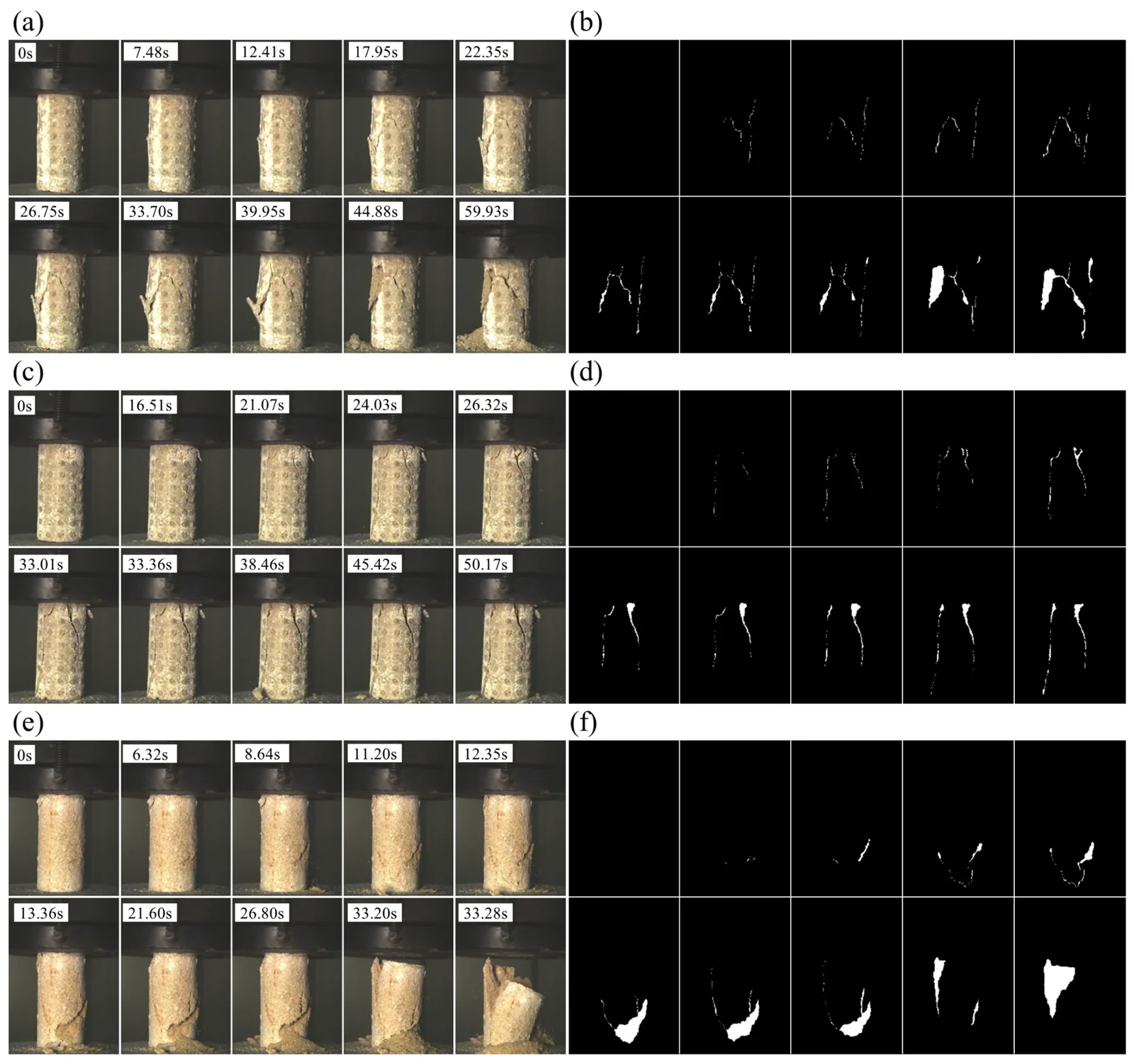
Crack evolution process and binarized crack identification for three MICP-cemented sand columns under compressive loading: (**a**,**b**) Compression 1; (**c**,**d**) Compression 2; (**e**,**f**) Compression 3.

Figure 16a–f presents the crack evolution process and corresponding binarized crack maps for three MICP-cemented sand columns under uniaxial compression. It can be observed that, first, at the middle to late stages of loading, distinct inclined shear bands develop, with their overall orientation forming an angle of approximately 45° to the loading direction, accompanied by local bulging and spalling of the specimens. Second, the binarized images show multiple crack branches and local coalescence, for example in specimen Compression 1 (Figure 16b), indicating that failure does not occur along a single planar shear surface; instead, once a primary shear band forms, several secondary cracks initiate and link up with it. This multi-crack pattern suggests the presence of numerous locally weakly cemented zones and reflects crack deflection as cracks bypass grain-scale obstacles, followed by the coalescence of multiple cracks into a through-going fracture. Overall, the experimental and numerical results show qualitative agreement in the dominant crack inclination and connectivity, although the local crack branches and tortuous paths vary across individual specimens.

It should be emphasized that the mesoscale model developed in this study is based on a two-dimensional plane-stress assumption and does not explicitly account for three-dimensional effects such as pore connectivity in the thickness direction, particle interlocking and rearrangement, or out-of-plane crack twisting and branching. As a result, the representation of post-peak “fragmentation and reloading”, multi-crack interaction, and complex three-dimensional splitting patterns is necessarily simplified. Nevertheless, the model successfully captures the characteristic mechanical response types and dominant failure modes under all three loading conditions. These predictions are consistent with the observations from high-speed imaging and crack identification, indicating that, within the scope of the present study, the 2D model provides a reasonable description of interface-governed failure and the role of the multiphase structure in MICP-cemented sand.

### 3.2. Differences in Failure Modes of the Multiphase MICP Structure Under Different Loading Conditions

By comprehensively analyzing the three representative loading conditions, namely uniaxial tension, direct shear, and uniaxial compression, the distinct failure modes of MICP-cemented sand under different stress states can be clearly identified.

Under uniaxial tension, failure is governed by the maximum principal tensile stress and manifests as a typical interface-controlled opening-mode brittle fracture. The crack path is approximately perpendicular to the loading direction, and the residual strength after failure is very low.

Under direct shear, the failure process exhibits a combined tensile-shear character. Crack initiation is mainly controlled by the principal tensile stress, whereas crack propagation is subsequently governed by the maximum shear stress, leading to the formation of a tortuous shear band along the shear plane. The response is quasi-brittle, dominated by interface cracking with frictional residual strength.

Under uniaxial compression, failure is controlled by the maximum shear stress, with inclined shear bands forming at an angle of about 45° to the loading direction. Friction and interlocking between particles provide significant residual load-carrying capacity after the peak, resulting in a quasi-brittle-frictional mixed failure mode.

Despite the differences in macroscopic failure patterns, the underlying mesoscale fracture processes follow a common mechanism: cracks preferentially initiate and propagate in mechanically weak regions, namely within the CaCO_3_ precipitates and along the CaCO_3_-sand interfaces, while the sand grains themselves remain essentially intact. The weak-interface effect and the heterogeneous nature of the MICP at the mesoscale fundamentally control the locations of crack initiation, the crack propagation paths, and ultimately the observed differences in macroscopic failure modes.

### 3.3. Failure Mechanism

**Figure 17:**
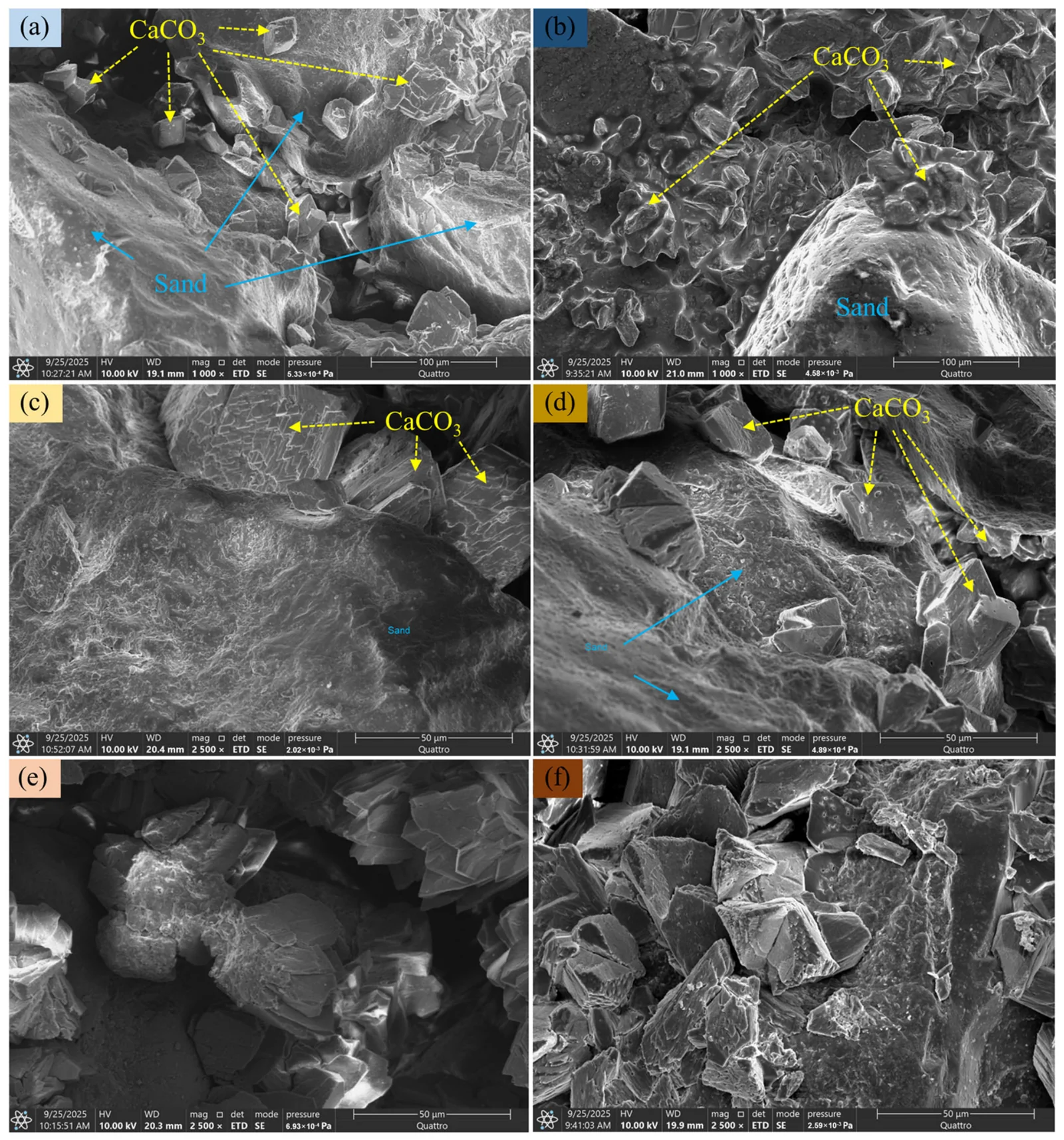
SEM images of fracture surfaces of MICP-cemented sand. (**a**–**d**) Interfacial debonding between MICP precipitates and sand grains, with intact grain surfaces and detached CaCO_3_ fragments; (**e**,**f**) Brittle fracture and heterogeneous distribution of MICP precipitates, showing plate-like and blocky fragments in locally concentrated cementation zones.

To verify the failure mechanisms of MICP-cemented sand under uniaxial tension, direct shear, and uniaxial compression at the microscopic scale, SEM observations were performed on the fracture surfaces of failed specimens, as shown in Figure 17. The observed morphologies provide direct evidence for interpreting the underlying mechanisms.

From the viewpoint of interfacial bonding, Figure 17a–d show that the sand grain surfaces on the fracture plane are generally smooth and intact, without obvious signs of tensile splitting or crushing. In contrast, a large amount of blocky, lamellar, or debris-like MICP precipitates remain around the grains. This indicates that cracks preferentially propagate along the interfaces between the precipitates and sand grains, and that failure is mainly controlled by interfacial debonding rather than by the fracture of the sand grains themselves or the uniform failure of a continuous cemented layer. This observation is highly consistent with the numerical prediction that CZM interface elements fail preferentially in the model.

Regarding the brittle fracture of the precipitates, Figure 17e,f show regions where the precipitates appear as stacked lamellae or blocks; locally, relatively flat facets, cleavage surfaces, and fine fragments can be observed, which are typical features of brittle fracture. This suggests that, in stress concentration zones, the MICP precipitates themselves undergo local cracking or crushing. Such fragmentation weakens the local bridging effect and facilitates faster coalescence of interface cracks.

Regarding the heterogeneity of the precipitates, an overall inspection of Figure 17a–f shows that the coverage of the precipitates on sand-grain surfaces and the filling of pore spaces are highly non-uniform and discontinuous. Some regions are densely cemented, whereas others are sparsely cemented or even uncemented. These weakly cemented zones act as natural stress concentrators and low-strength paths, where microcracks are prone to initiate and preferentially propagate.

In summary, combining the numerical simulations with the SEM observations, a unified failure mechanism for MICP-cemented sand can be formulated as follows: under external loading, microcracks first initiate in weakly cemented regions or at stress concentration sites; subsequently, cracks propagate mainly along the lower-strength interfaces between the precipitates and sand grains, bypassing the grains; during this process, local brittle fracture of the precipitates provides “shortcuts” that promote crack linkage. Ultimately, multiple microcracks coalesce into macroscopic through-going fractures, leading to global failure of the cemented structure.

## 4. Conclusions

In this study, a classical two-phase random aggregate placement scheme was extended into a multiphase random particle placement algorithm tailored for MICP-cemented sand, and combined with a CZM-based finite element framework to construct a mesoscale fracture model that explicitly captures the multiphase precipitation features. Together with systematic mechanical tests, this model was used to investigate the deformation and failure behavior of MICP-cemented sand under different stress states. The main conclusions are as follows:

(1) The finite element approach developed in this work, which integrates the extended multiphase random particle placement algorithm with a cohesive zone model, can effectively represent the heterogeneous multiphase meso-structure of MICP-cemented sand and capture the main response characteristics and crack propagation behavior under uniaxial tension, direct shear, and uniaxial compression. Compared with conventional two-phase aggregate models, the proposed algorithm explicitly places sand grains and multiple CaCO_3_ polymorphs with prescribed volume fractions, size ratios, and shapes, by first generating a DEM-type packing and then importing it into a CZM-based FE mesh, thereby providing a generic workflow that links particle-scale packing with interface-dominated fracture simulation.
(2) The sand particle distribution is a key mesoscale factor governing the mechanical response and fracture evolution of MICP-cemented sand. The simulations show that the sand particle distribution has limited influence on the linear elastic segment and peak strength of the stress–strain curves, but significantly affects the post-peak failure stage. Crack deflection and bypassing caused by particle obstruction mainly affect crack coalescence and post-peak softening.
(3) The macroscopic failure modes of MICP-cemented sand differ markedly under different stress states. Tensile failure is characterized by interface-controlled brittle fracture, with cracks perpendicular to the loading direction. Shear failure exhibits quasi-brittle behavior, with a damage band forming along the shear plane. Compressive failure shows a quasi-brittle-frictional mixed mode, with inclined shear bands at approximately 45° to the loading direction.
(4) Despite the differences in macroscopic patterns, the mesoscale failure mechanisms under the three loading conditions share a common feature: microcracks initiate in weakly cemented zones or stress concentration regions, then propagate mainly along the interfaces between precipitates and sand grains and bypass the grains; local brittle fracture of the precipitates accelerates crack linkage, and multiple microcracks eventually coalesce into macroscopic through-going fractures, leading to global structural failure.

Overall, the proposed multiphase particle placement-CZM finite element framework deepens the understanding of the mechanical behavior and fracture mechanisms of MICP-cemented sand and provides a mesoscale framework for analyzing interface-dominated fracture in MICP-cemented materials.

## Figures and Tables

**Table 1:** Basic material parameters of MICP-cemented sand.

Material	Elastic Modulus (GPa)	Poisson’s Ratio	Density (g/cm^3^)
Calcite	73.2	0.3	2.71
Aragonite	140	0.32	2.95
Vaterite	58	0.28	2.64
MICP amorphous phase	20	0.2	2.00
Sand grain [49,51]	40	0.15	2.70

**Table 2:** CZM parameters for the MICP amorphous phase and amorphous-particle interfaces.

Material	Initial Stiffness (GPa/μm)	Tensile Strength (MPa)	Tensile Fracture Energy (N/m)	Compressive Strength (MPa)	Compressive Fracture Energy (N/m)	Shear Strength (MPa)	Shear Fracture Energy (N/m)
MICP amorphous phase	120	0.16	2	1.1	8.0	0.12	0.9
Amorphous-particle interface	100	0.15	1.8	1	7.6	0.11	0.86

## Data Availability

The original contributions presented in this study are included in the article. Further inquiries can be directed to the corresponding authors.
